# Humanized mouse liver reveals endothelial control of essential hepatic metabolic functions

**DOI:** 10.1016/j.cell.2023.07.017

**Published:** 2023-08-09

**Authors:** Eleanna Kaffe, Manolis Roulis, Jun Zhao, Rihao Qu, Esen Sefik, Haris Mirza, Jing Zhou, Yunjiang Zheng, Georgia Charkoftaki, Vasilis Vasiliou, Daniel F. Vatner, Wajahat Z. Mehal, AlcHepNet, Yuval Kluger, Richard A. Flavell

**Affiliations:** 1 Department of Immunobiology, Yale School of Medicine, New Haven, CT, 06520, USA.; 2 Department of Pathology, Yale School of Medicine, New Haven, CT, 06520, USA.; 3 Computational Biology and Bioinformatics Program, Yale University, New Haven, CT, 06511, USA.; 4 Department of Environmental Health Sciences, Yale School of Public Health, Yale University, New Haven, CT, 06520, USA.; 5 Department of Internal Medicine, Section of Endocrinology, Yale School of Medicine, New Haven, CT, 06520, USA.; 6 Department of Internal Medicine, Section of Digestive Diseases, Yale University, New Haven, CT, 06520, USA.; 7 Veterans Affairs Medical Center, West Haven, CT, 06516, USA.; 8 NIAAA Alcoholic Hepatitis Consortia.; 9 Program of Applied Mathematics, Yale University, New Haven, CT, 06511, USA.; 10 Howard Hughes Medical Institute, Yale School of Medicine, New Haven, CT, 06519, USA.

**Keywords:** Humanized liver, liver sinusoidal endothelial cells (LSECs), cholesterol, bile acid conjugation, WNT2, FZD5, stellate cells, fibrosis, lipidomics, non-alcoholic fatty liver disease (NAFLD)

## Abstract

Hepatocytes, the major metabolic hub of the body, execute functions that are human-specific, altered in human disease and currently thought to be regulated through endocrine and cell-autonomous mechanisms. Here, we show that key metabolic functions of human hepatocytes are controlled by non-parenchymal cells (NPCs) in their microenvironment. We developed mice bearing a human hepatic tissue, composed of human hepatocytes and NPCs, including human immune, endothelial and stellate cells. Humanized livers reproduce human liver architecture, perform vital human-specific metabolic/homeostatic processes and model human pathologies, including fibrosis and non-alcoholic fatty liver disease (NAFLD). Leveraging species-mismatch and lipidomics, we demonstrate that human NPCs control metabolic functions of human hepatocytes in a paracrine manner. Mechanistically, we uncover a species-specific interaction whereby WNT2 secreted by sinusoidal endothelial cells controls cholesterol uptake and bile acid conjugation in hepatocytes through receptor FZD5. These results reveal the essential microenvironmental regulation of hepatic metabolism and its human-specific aspects.

## In Brief

A comprehensive human liver tissue was established in a mouse host that consists of all human-relevant parenchymal and non-parenchymal cell types and mimics the cellular composition, histological architecture, and functional properties of a human liver. This highly human-relevant murine model allows investigation of human-specific metabolic features and liver cell type interactions.

## Graphical Abstract

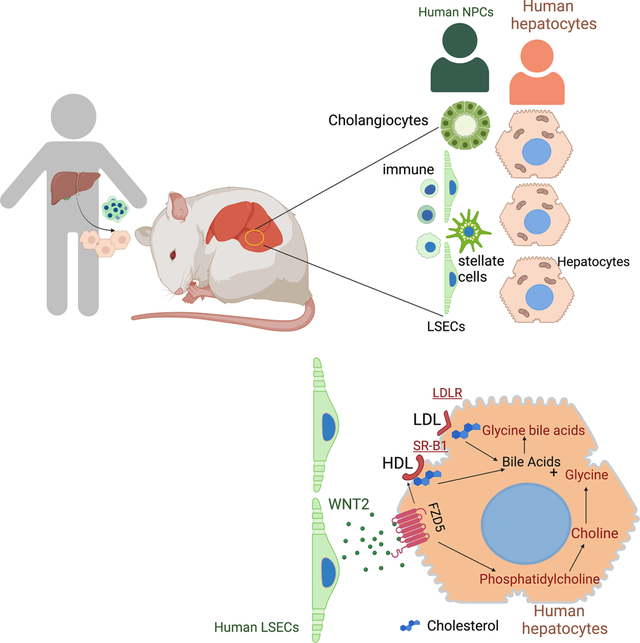

## INTRODUCTION

The human liver is a crucial organ, which orchestrates numerous essential functions^1^. Despite being constantly exposed to numerous damaging insults, it retains its functionality through many mechanisms including an extensive capacity for homeostatic regeneration^2^. This is essential for the uninterrupted metabolic function of hepatocytes, which are a primary site for vital processes, such as lipid metabolism, xenobiotic and drug detoxification, vitamin biotransformation, nutrient storage and acute phase response^1^. In chronic liver diseases, hepatocyte malfunction represents a major cause of morbidity and of a mortality of an estimated 2 million deaths per year^3,4^. Thus, understanding how hepatocyte function is regulated in the dynamic environment of the liver is crucial to understanding the mechanisms underlying hepatocyte dysfunction in the setting of chronic liver disease.

The need to understand these issues is most acute in humans as core metabolic functions of the human liver are fundamentally different from those of rodents. Major differences exist in key biosynthetic functions important for maintaining whole body homeostasis, such as the metabolism of xenobiotics and drugs^5^, the detoxification of cholesterol to bile acids, bile acid conjugation and lipoprotein production^6^. For example, humans have a different bile acid profile from that of mice, with a high abundance of chenodeoxycholic acid (CDCA) and lack of muricholic acids, which are respectively a strong agonist and physical antagonists of the nuclear receptor FXR^7,8^. Notably, pharmacological agonism of FXR with obeticholic acid (OCA) in preclinical studies in rodents led to a significant decrease of low-density lipoprotein (LDL) cholesterol^9 10^, whereas OCA administration to healthy volunteers^11^ and to non-alcoholic fatty liver disease (NAFLD) patients in clinical trials^12^ had the *opposite* effect: it led to a substantial increase of LDL cholesterol in the blood. These findings exemplify the profundity of species specificities in hepatic metabolism and underscore the limitations of mouse models as a tool to understand the metabolic function of the human liver.

A promising strategy to study the function of human cell types *in vivo* is mouse humanization. Based on elegant prior technologies that enabled the humanization of hepatocytes (using *Fah*^−/−^ mice)^13^ and of the immune system (MISTRG6 mice)^14,15^, we recently established a humanized mouse model that comprises both human hepatocytes and human immune cells in the same mouse host^16^. Still, in current liver humanization systems, the remainder of the hepatic cell types that comprise NPCs, including liver sinusoidal endothelial cells (LSECs), hepatic stellate cells, cholangiocytes and portal fibroblasts, are of murine or uncharacterized origin. This is an important limitation, since stellate cells and endothelial cell types closely interact with hepatocytes, are potentially important for liver morphogenesis^17^, function and repair upon damage^18^ and are also involved in the development of fibrosis in the context of liver diseases^19^. Thus, current humanization technologies are not amenable to study how human hepatocytes interact with their microenvironment and the functional impact of this interaction.

We hypothesized that comprehensive humanization of the hepatic tissue in a mouse host may be feasible after considering the developmental origin of each liver cell type in humans and the developmental aspects of humanization of the MISTRG6 technology. MISTRG6 mice bear alleles encoding human M-CSF (CSF1), IL-3, SIRPα, thrombopoietin, GM-CSF (CSF2) and IL-6, knocked into their respective mouse loci on an immunodeficient *Rag2*^−/−^*Il2rg*^−/−^ genetic background. Transplantation of human CD34^+^ hematopoietic stem cells into these mice leads to a robust development of human lymphoid and myeloid cells^14,15^. Previous studies have shown that CD34 is expressed in non-hematopoietic hepatic tissue cell types during fetal liver development^20,21,22,23^. This suggests that CD34^+^ Fetal Liver Cells (FLCs) used for mouse immune cell humanization may contain precursors of non-hematopoietic NPCs. Based on this notion, we hypothesized that the transplantation of human CD34^+^ FLCs into MISTRG6 mice may support humanization of additional non-immune hepatic lineages.

Based on this hypothesis, here, we developed humanized livers comprising the majority of the human hepatic tissue cell types. We employed this technology to understand whether the metabolic function of human hepatocytes is internally-regulated or subject to paracrine control by their microenvironment. Our results reveal that important metabolic functions of human hepatocytes are controlled by NPCs and identify cellular and molecular pathways that can be therapeutically targeted to prevent hepatocyte malfunction in human liver disease.

## RESULTS

### Development of a human hepatic tissue in a mouse host

**Figure 1.**
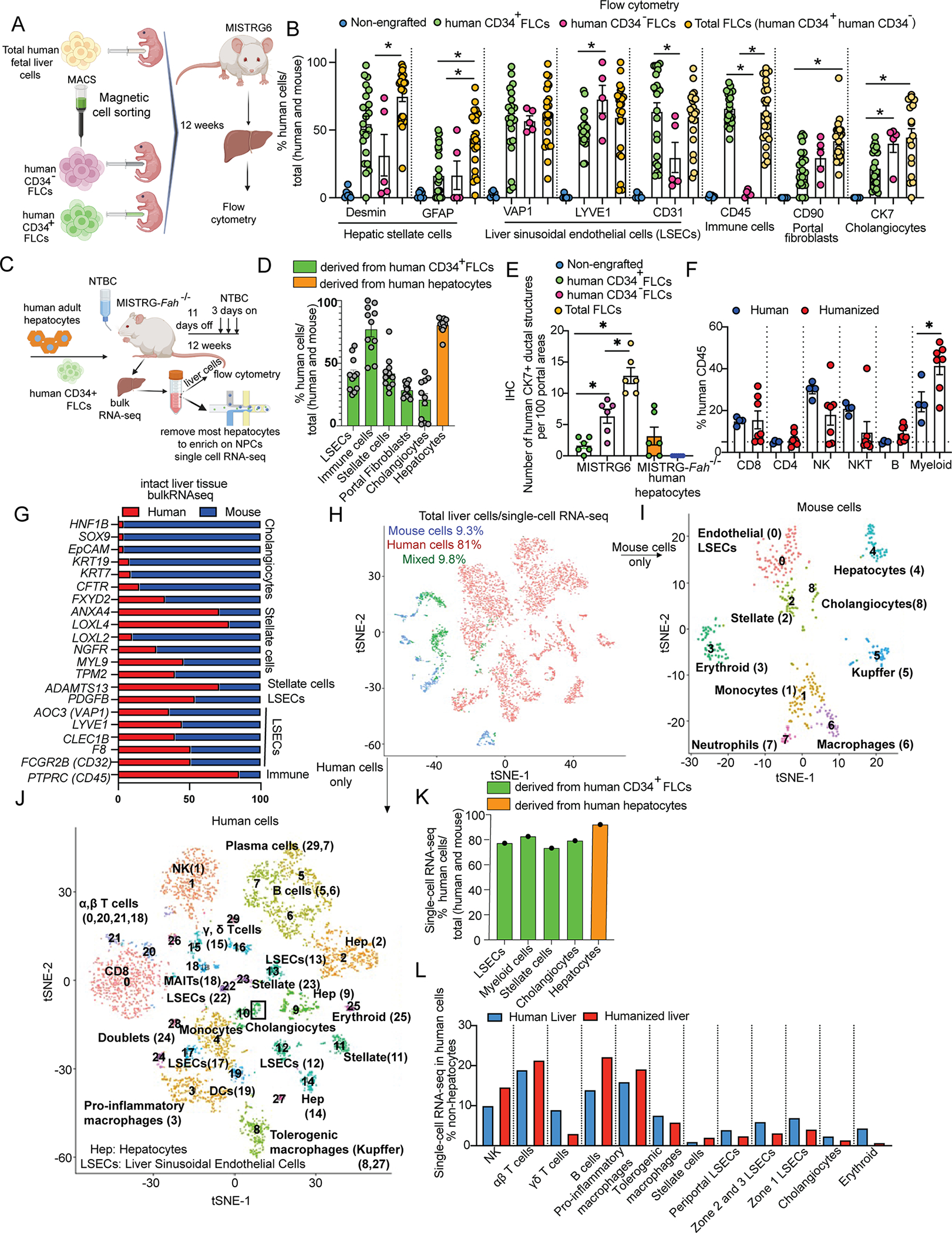
Development of a human hepatic tissue in a mouse host. (**A**) Neonatal MISTRG6 mice were engrafted intrahepatically with total FLCs or, after MACS, with human CD34^+^ (more than 95% purity) or CD34^−^ FLCs (one donor with the highest humanization had 83% purity and the other with the lowest humanization had 91% purity). (**B**) Flow cytometry in liver cells using specific anti-mouse or anti-human antibodies. (**C**) Adult MISTRG-*Fah*^−/−^ mice were engrafted with human CD34^+^ FLCs and human hepatocytes. Liver was collected for bulk RNA-seq or was digested for liver cells isolation. (**D**) Quantification of the humanization of all major liver cell subpopulations by flow cytometry. (**E**) Quantification of the human CK7^+^ biliary structures by IHC. (**F**) Quantification of the humanization of immune cell subtypes in the humanized liver and in control healthy human liver tissue. Percentage of human NK cells (CD56^+^), NKT cells (CD56^+^CD3^+^), B cells (CD19^+^), myeloid cells (CD33^+^) among CD45^+^ cells. (**G**) Bulk RNA-seq in liver tissue. Relative abundance of mouse and human orthologous genes that are unique markers of cholangiocytes, LSECs, immune and stellate cells. From the same mouse we isolated liver cells for single cell-RNA sequencing. (**H-J**) t-SNE plots for human and mouse cells after single-cell RNA-seq in liver cells enriched for NPCs. (**K**) Percentage of human cells over total cells (mouse and human) for each major liver cell type, calculated by single-cell RNA-seq in the humanized liver. (**L**) Relative abundance of human cell counts in the human and in the humanized liver for all major non-hepatocyte liver cell types. The data for the human liver were retrieved by published datasets^24^. Cartoons were made using BioRender. See also Figures S1–S4. Each dot in the graphs is a biological replicate from at least 2 independent experiments. The cell extraction protocol used in Figure 1B, D, H–K was suboptimal for the biliary tree hence the estimated percentage of human cholangiocytes is not accurate. Data represent mean ± SEM. ns, non-significant; *p < 0.05.

To examine whether we could humanize liver NPCs, we transplanted human CD34^+^ FLCs, human CD34^−^ FLCs or their combination intra-hepatically in 2-day-old MISTRG6 mice (Figure 1A). At 12 weeks post-transplantation, we analyzed the livers by flow cytometry with a panel of human and mouse-specific markers for LSECs, stellate cells, immune cells, portal fibroblasts and cholangiocytes (Figure S1). We found that after engraftment of the human CD34^+^ fraction alone, ~50% of LSECs and Desmin^+^ stellate cells and ~20% of cholangiocytes and portal fibroblasts that were recovered after liver digestion and dead cell removal were human (Figure 1B). Combined engraftment of human CD34^+^ and human CD34^−^ FLCs further increased the humanization of cholangiocytes (Figure 1B, E) and portal fibroblasts (but did not affect the presence of the other NPCs), as compared to the human CD34^+^ fraction alone (Figure 1B). These results demonstrate that MISTRG6 mice support the humanization of a broad spectrum of liver NPCs thus enabling the study of the effect of these human populations on human hepatocyte function.

Next, we aimed to develop a system in which we can study the functional interaction of human hepatocytes with human NPCs *in vivo*, by humanizing these cell populations in the same mouse host. For this purpose, we generated the MISTRG-*Fah*^−/−^ mice which is a well-established system of hepatocyte humanization^16^. The MISTRG-*Fah*^−/−^ mice in which we simultaneously engrafted human CD34^+^ FLCs and human hepatocytes (Figure S2A, Figure 1C) and found that the humanization of immune cells, LSECs and stellate cells in their liver reached levels similar to those observed in MISTRG-*Fah*^−/−^ mice transplanted with mouse hepatocytes or MISTRG6 controls; thus, this process was not affected by NTBC/Fah-deficiency-driven liver damage (Figure S2B–D, F). We also observed an overall increased humanization of the blood immune cells of MISTRG-*Fah*^−/−^ mice transplanted with human hepatocytes as compared to mouse hepatocyte-recipient controls (Figure S2E). These results show that hepatocytes and NPCs can be successfully co-humanized in the same mouse host.

To further assess the potential for humanization of all major liver cell types, we developed an additional model for establishing humanized livers, independent of the *Fah*^−/−^ system. We devised a strategy of controlled replacement of mouse by human hepatocytes in MISTRG6 mice, by inhibiting the ability of mouse hepatocytes to proliferate with retrosine (a cell cycle inhibitor), and by inducing mouse hepatocyte death through treatments with acetaminophen (APAP) before human hepatocyte transplantation and injection of an anti-mouse Fas antibody after hepatocyte transplantation. We named this model MISTRG6-**RAF** (after **R**etrosine-**A**PAP-anti-mouse **F**AS) (Figure S2A). We found comparable hepatocyte humanization in MISTRG6-RAF mice to that of the MISTRG-*Fah*^−/−^ model (Figure S2G–H). Upon simultaneous engraftment of human CD34^+^ FLCs, MISTRG6-RAF mice displayed a similar humanization of immune cells, endothelial cells and stellate cells to that of MISTRG-*Fah*^−/−^ mice (Figure S2B–D). MISTRG6-RAF mice do not require cycling administration of NTBC, a tyrosine metabolism inhibitor for both human and mouse cells, and they appear to be more robust with better survival than MISTRG-*Fah*^−/−^ mice. These results establish the MISTRG6-RAF model as a viable alternative approach for the concurrent humanization of hepatocytes and NPCs in the liver of the same mouse host.

To understand the precise identity of human cells in a humanized liver, we performed an in-depth characterization of the humanization aspects of MISTRG-*Fah*^*−/−*^ mice. For this purpose, we generated independent cohorts of MISTRG-*Fah*^−/−^ mice which we transplanted with human hepatocytes along with human CD34^+^ FLCs (Figure 1C). Flow cytometry analyses in the liver 12 weeks after human cell transplantation confirmed the partial humanization of all major hepatic cell populations and quantitated its extent (Figure 1D). Considering that the liver digestion protocol employed for flow cytometry is suboptimal for cholangiocyte extraction, we also performed immunohistochemistry (IHC) for human CK7. We found the highest number of human bile ducts in MISTRG6 mice engrafted with combined human CD34^+^ and CD34^−^ FLCs and absence of human bile ducts in MISTRG-*Fah*^−/−^ mice engrafted with human hepatocytes only (Figure 1E). Detailed characterization of immune subsets in the humanized livers by flow cytometry showed that they have a composition similar to that of normal human liver tissue obtained from partial hepatectomy (Figure 1F). We corroborated the quantitative flow cytometry results for all major NPC populations by measuring the percentage of human versus mouse gene expression for a set of endothelial, stellate, cholangiocyte and immune markers, determined by RNA-seq analysis (Figure 1G). Single-cell RNA-seq analysis of NPC-enriched liver preps from humanized MISTRG-*Fah*^−/−^ mice, validated that the humanization of the liver is extensive (Figure 1H) and involves all major hepatic tissue cell populations, revealing the presence of 29 clusters of human cells and of 8 clusters of mouse cells (Figure 1I–J). We compared the transcriptional profile of each individual human cell and of each cluster with cell type-specific signatures (via metagenes) based upon a single cell atlas of the human liver^24^ (Figures S3, S4). These analyses uncovered additional immune populations, including plasma cells, gamma-delta T-cells, MAIT cells (Figure S3C–D) as well as two distinct macrophage populations, monocytes and dendritic cells (Figure S3E–G). We also confirmed the presence of human stellate cells, portal fibroblasts and cholangiocytes (Figure S4B–C). Moreover, we observed that hepatocytes (Figure S4A) and endothelial cells (Figure S4D–H) formed several different clusters, similar to those in a healthy human liver single cell atlas^24^, showing that hepatocyte and LSEC diversity reflects the unique zonated architecture of the liver tissue. Importantly, not only the composition and gene signatures but also the relative abundance of cell types in the humanized liver matches that of the human liver^24^ (Figure 1L). Thus, we established the major human hepatic cell populations in a mouse host.

### Humanized livers recapitulate human liver architecture.

**Figure 2.**
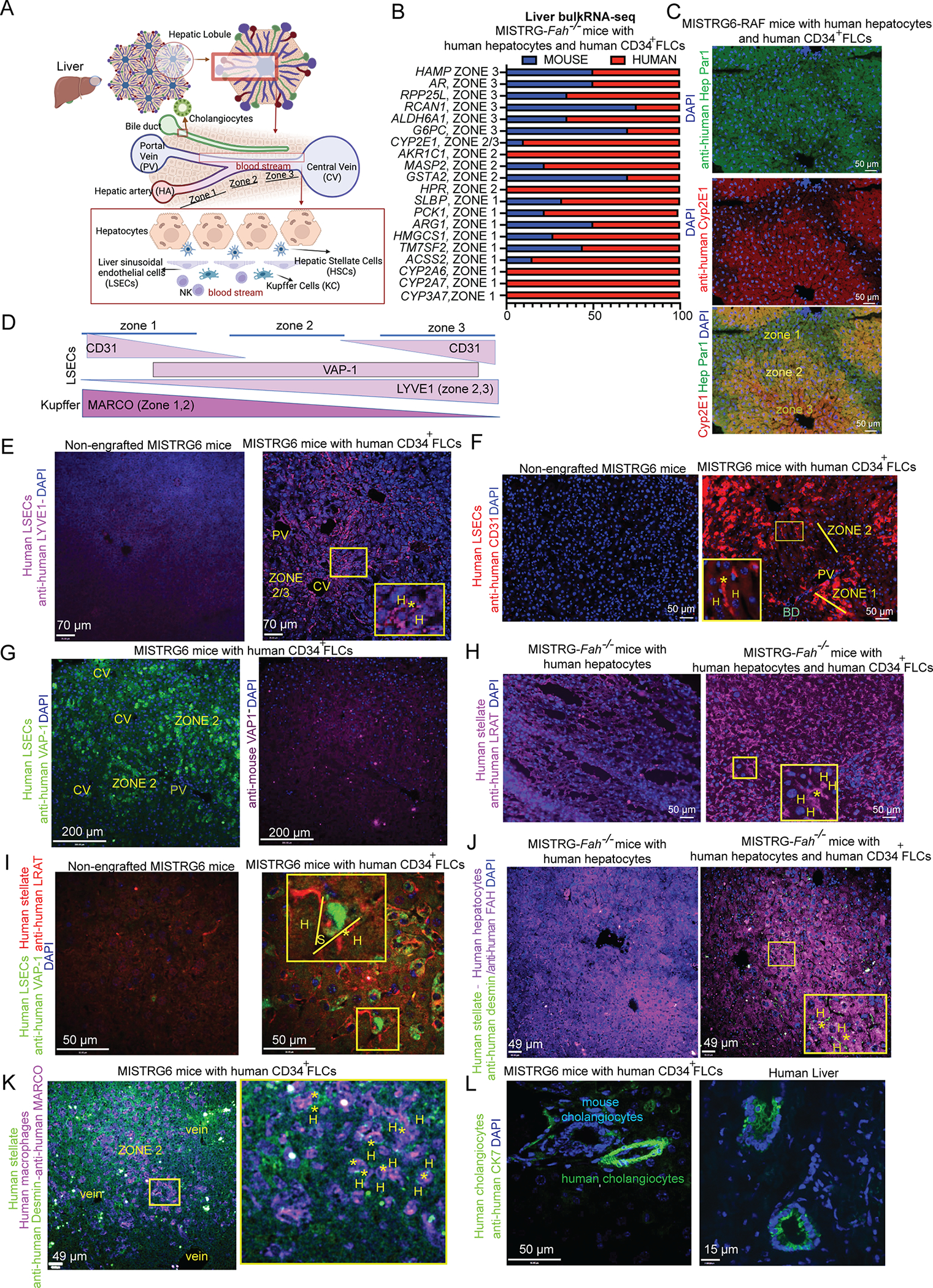
Spatial distribution of human cells in humanized livers. **(A)** Liver architecture and organization of liver cell types in the hepatic lobule. Cartoon was made using Biorender. **(B)** Bulk RNA-sequencing in the liver of humanized MISTRG-*Fah*^*−/−*^ mice. Relative abundance of mouse and human orthologous hepatocyte predominant genes. **(C)** Immunofluorescence for human Cyp2E1 (zone 2, 3 protein) and human Hep Par in humanized MISTRG6-RAF mice. The antigen for Hep Par 1 antibody is the urea cycle enzyme CPS1 (zone 1, 2 protein). **(D)** Expression of LSEC and Kupffer cell markers in different zones of the hepatic lobule, based upon the human protein atlas and literature^24^. (**E-G)** Human-specific LSEC markers (LYVE-1, VAP-1, CD31) and their expression across liver zones in MISTRG6 mice engrafted with human CD34^+^ FLCs or non-engrafted controls. Anti-human CD31 may stain a few human macrophages. Here it is used to validate that human CD31 is not expressed in zone 2 cells. **(H)** Immunostaining against human LRAT **(I-K)** Immunostainings for human LRAT, Desmin, FAH, VAP-1 and MARCO. Examples of the human cell types of interest, having the proper localization between hepatocytes (H) in sinusoids (S), are indicated with an asterisk (*). **(L)** Immunostaining for human CK7 in humanized liver and healthy human liver sections. All mice were analyzed 12 weeks after human CD34^+^ FLC (E-G, I, K-L) and human hepatocyte transplantation **(C, H, J).** Mice that were not engrafted with human CD34^+^ FLCs served as controls for the human specificity of antibodies. See also Figure S5.

Next, we focused on the spatial organization of human cell types within the humanized liver. First, we confirmed that the humanization of hepatocytes occurred throughout most of the organ’s space in both MISTRG-*Fah*^−/−^ and MISTRG6-RAF mice (Figure S2H). Since hepatocytes and the associated LSECs are organized into three anatomical and functional zones which are determined by oxygen availability^25^, we examined tissue zonation (Figure 2A). By performing RNA-seq analyses in whole liver tissue from MISTRG-*Fah*^−/−^ mice and determining the percentage of human versus mouse gene expression for a set of zone-specific marker genes^24^, we detected robust humanization of hepatocytes across all three zones of the liver (Figure 2B). At the single-cell level, we calculated zone-specific metagenes based on a human liver atlas^24^ and found that each hepatocyte cluster of the humanized liver corresponds to a specific zone (Figure S4A). Similarly, we observed that each LSEC cluster matches to a specific zone (Figure 1L, S4D–F). We confirmed the zonation of human hepatocytes *in situ* in both MISTRG-*Fah*^−/−^ and MISTRG6-RAF livers by immunostainings for the zone-specific markers CYP2E1 (zones 2, 3) and Hep Par-1 (zones 1, 2) (Figures 2C). We confirmed the zonation of human LSECs *in situ* by immunostaining for the zone- and human-specific markers LYVE1^26^, CD31 and VAP-1 (Figures 2D–G). By staining for CD34, known to be expressed in the LSECs in the late embryonic and fetal periods and lost in neonates and adults^27^, we validated its absence from LSECs (Figure S5A). On the other hand, upon maturation of the liver from the fetus to adulthood, expression of CD34 in the central and portal vein endothelial cells increases. Thus, we validated CD34 expression in the venous endothelial cells of central veins (Figure S5B). These results indicate that FLC-derived human LSECs are like the mature-adult ones. We also confirmed the presence of human macrophages in all three zones (as expected) by immunostainings for human-specific CD68 (Figure S5C).

To understand if the zonation of human hepatocytes and the associated sinusoidal endothelium reflects the successful development of a human liver architecture (Figure 2A), consisting of hepatocyte plates segregated firstly by stellate cells and secondly by sinusoids lined by LSECs and Kupffer cells, we examined the location of each cell type respective to its adjacent cell type in the hepatic lobule. By staining for human desmin/LRAT we found that human stellate cells are widespread in the lobule (Figure 2H) and located between human LSECs and human hepatocytes along sinusoids (Figure 2I–J) as well as between human Kupffer cells (MARCO^+^) and hepatocytes (Figure 2K) similarly to their location in the healthy human liver. Immunostaining for human CK7, revealed that human cholangiocytes form human bile duct structures that are adjacent to mouse bile ducts in the portal area (Figure 2L), which is their appropriate location. These results demonstrate that the humanized livers that we generated successfully recapitulate to a substantial degree both the cellular composition and the tissue architecture of the normal human liver.

### Human liver cells possess key cell-type specific functional capacities *in vivo*.

**Figure 3.**
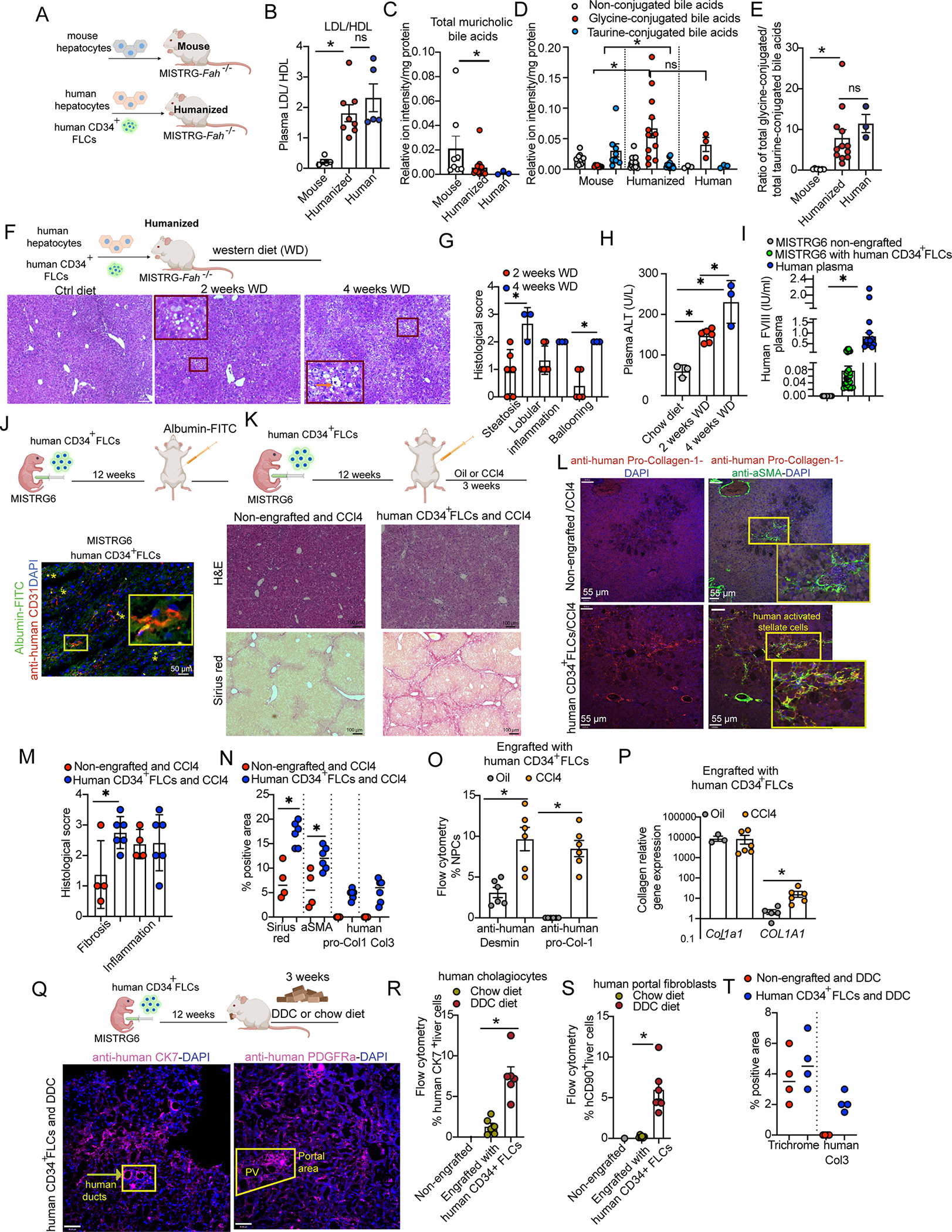
Human liver cells possess key cell type-specific functional capacities *in vivo.* **(A)** Adult MISTRG-*Fah*^−/−^ mice were engrafted with human CD34^+^ FLCs and human hepatocytes (*humanized mouse*) or murine FAH-WT hepatocytes only (*mouse*). **(B)** Plasma LDL/HDL cholesterol ratio. **(C-E)** Bile acids in the hepatocyte fraction measured with HPLC-MS/MS. **(F-G)** H&E and histological score in humanized MISTRG-*Fah*^−/−^ mice fed with a western diet or standard chow diet for 2 or 4 weeks. Orange arrow shows ballooning. **(H)** Plasma ALT levels. **(I)** Human Factor VIII in the plasma of MISTRG6 mice and healthy volunteers. **(J)** Uptake of an FITC-albumin conjugate (green) by human endothelial cells (CD31^+^, in red) in the liver of humanized MISTRG6 mice. Yellow asterisks indicate areas of colocalization. The cropped images in yellow rectangles show areas of colocalization at a higher magnification. **(K, M)** Induction of fibrosis by CCl4 in MISTRG6 mice engrafted with human CD34^+^ FLCs and non-engrafted controls. H&E and Sirius red staining and the respective histological score are shown. **(L)** Immunofluorescence for aSMA (human and mouse, dual specificity, in green) and human pro-collagen-1 (in red). Yellow rectangles indicate human activated stellate cells in yellow. **(N)** Quantification of Sirius red, human collagen-3 and human pro-collagen-1 positive area using Image-J. **(O)** Quantification of human Desmin and human pro-collagen-1-expressing NPCs by flow cytometry. **(P)** Gene expression of mouse *Col1a1* and human *COL1A1*, quantitated by RT-qPCR. **(Q)** Induction of ductular reaction in MISTRG6 mice engrafted with human CD34^+^ FLCs and non-engrafted controls. The mice were fed ad-libitum with 0.01% DDC or chow diet for 3 weeks. Immunofluorescence for human CK7 and human PDGFRa in purple. **(R, S)** Quantification of human cholangiocytes (CK7^+^ cells) and human portal fibroblasts (CD45^−^CD90^+^ cells) in the liver by flow cytometry. **(T)** Quantification of trichrome staining for total collagen and human collagen-3 positive area using Image-J. All cartoons were made using BioRender. See also Figure S5. Each dot in the graphs is a biological replicate; n>3 biological replicates from 2 independent experiments. Data represent mean ± SEM. ns, non-significant; *p < 0.05.

To assess the functionality of humanized livers, we generated MISTRG-*Fah*^−/−^ mice engrafted with human hepatocytes and human CD34^+^cells (Figure 3A) and first assessed the human specificity of lipoprotein synthesis and secretion a major function of liver as a whole organ. Human, but not mouse liver, lacks APOBEC-1 which converts ΑpoB-100 to ΑpoB-48^28^. Therefore, the amount of Αpo-B100 (an essential component of LDL) produced by the liver is low in mice and high in humans. Moreover, human liver is the major source of the cholesteryl ester transfer protein (CETP) that is absent in rodents^29^. CETP catalyzes the exchange of cholesterol esters and triglyceride between HDL and LDL^30^. Together, the lack of the *CETP* gene and the low levels of ΑpoB-100 in the circulation of rodents helps to explain their low LDL/HDL ratio. We found that the LDL/HDL ratio in the plasma of mice with a humanized liver is similar to that of humans, in sharp contrast to mouse controls (Figure 3B). To specifically examine the functionality of human hepatocytes within a humanized liver, we analyzed the production of bile acids, a key hepatocyte function with strong species differences. Humans, unlike mice, lack the Cyp2c70 cytochrome and therefore do not synthesize muricholic bile acids as mice do^31^. Also, humans have predominantly glycine-conjugated bile acids whereas mice have taurine-conjugated bile acids because of a different affinity of the BAAT conjugating enzyme for glycine or taurine between the two species^32^. We found that humanized livers have much lower levels of muricholic acids than control mice (Figure 3C) and display a ratio of glycine vs taurine-conjugated bile acids that is the same as that of the human liver (Figure 3D–E). Thus, these essential and species-specific metabolic functions of the liver in humanized mice operate as in humans. To assess whether our humanized system could model human NAFLD, we examined the response of humanized livers to a western diet. Strikingly, we found that western diet-fed humanized mice displayed major histological features of NAFLD, including Mallory bodies, ballooning (Figure S5D, Figure 3F) and zone 3 steatosis in human hepatocytes (Figure S5D–F, Figure 3F), as well as lobular inflammation (Figure 3G). All these features, along with liver damage markers (ALT), progressively increased from week-2 to week-4 of the dietary treatment and were absent in control humanized mice fed with a standard chow diet (Figure 3 F–H). These results show that in response to a common human liver damaging insult, a humanized liver can successfully recapitulate the complex pathophysiological processes that drive the development of human NAFLD.

We assessed the functionality of human NPCs within the humanized liver, starting with LSECs. We detected human Factor VIII in the plasma of humanized mice, an essential blood clotting factor that is produced and secreted by LSECs and other endothelial cells^33^ (Figure 3I). A major homeostatic function of LSECs is to scavenge macromolecules from the blood circulating in sinusoids. By injecting mice with a humanized liver with an FITC-albumin conjugate, we found that human LSECs efficiently take up this macromolecule from the sinusoidal area (Figure 3J). To test the functionality of human hepatic stellate cells we examined their ability to initiate matrix reconstitution upon liver damage. For this purpose, we treated MISTRG6 mice engrafted with human CD34^+^ FLCs with the hepatotoxin carbon tetrachloride (CCl4), an established model of stellate cell-driven liver fibrosis^19^. Mice engrafted with human CD34^+^ cells displayed more liver damage and a higher degree of fibrosis than non-engrafted mice (Figure 3K, M). Immunostaining, gene expression and flow cytometry analyses showed that human hepatic stellate cells expand upon CCl4-mediated damage, differentiate into human collagen-1-producing αSMA^+^ myofibroblasts and produce human collagen-3 (Figure 3L,N–P, Figure S5I) as it is observed in humans with liver fibrosis^34^. To test whether human cholangiocytes and human portal fibroblasts can expand in the context of cholangiopathies, we treated mice with 3,5-diethoxycarboncyl-1,4-dihydrocollidine (DDC). DDC induced chemical biliary tree damage and remodeling, a process entitled the ductular reaction, equally in engrafted with human CD34^+^ FLCs and non-engrafted MISTRG6 mice (Figure S5G, H). We found that human cholangiocytes increased upon damage in the engrafted mice (Figure 3Q, R). This process was accompanied by a wound healing response involving human portal fibroblast expansion (Figure 3Q, S) and human collagen-3 production (Figure S5I, Figure 3T). These results show that the humanized liver as a whole, and all the major human cell types therein perform key metabolic, homeostatic and regenerative/fibrotic processes and also recapitulate their human-specific aspects.

### Human hepatocyte metabolic profile is shaped by NPCs

**Figure 4.**
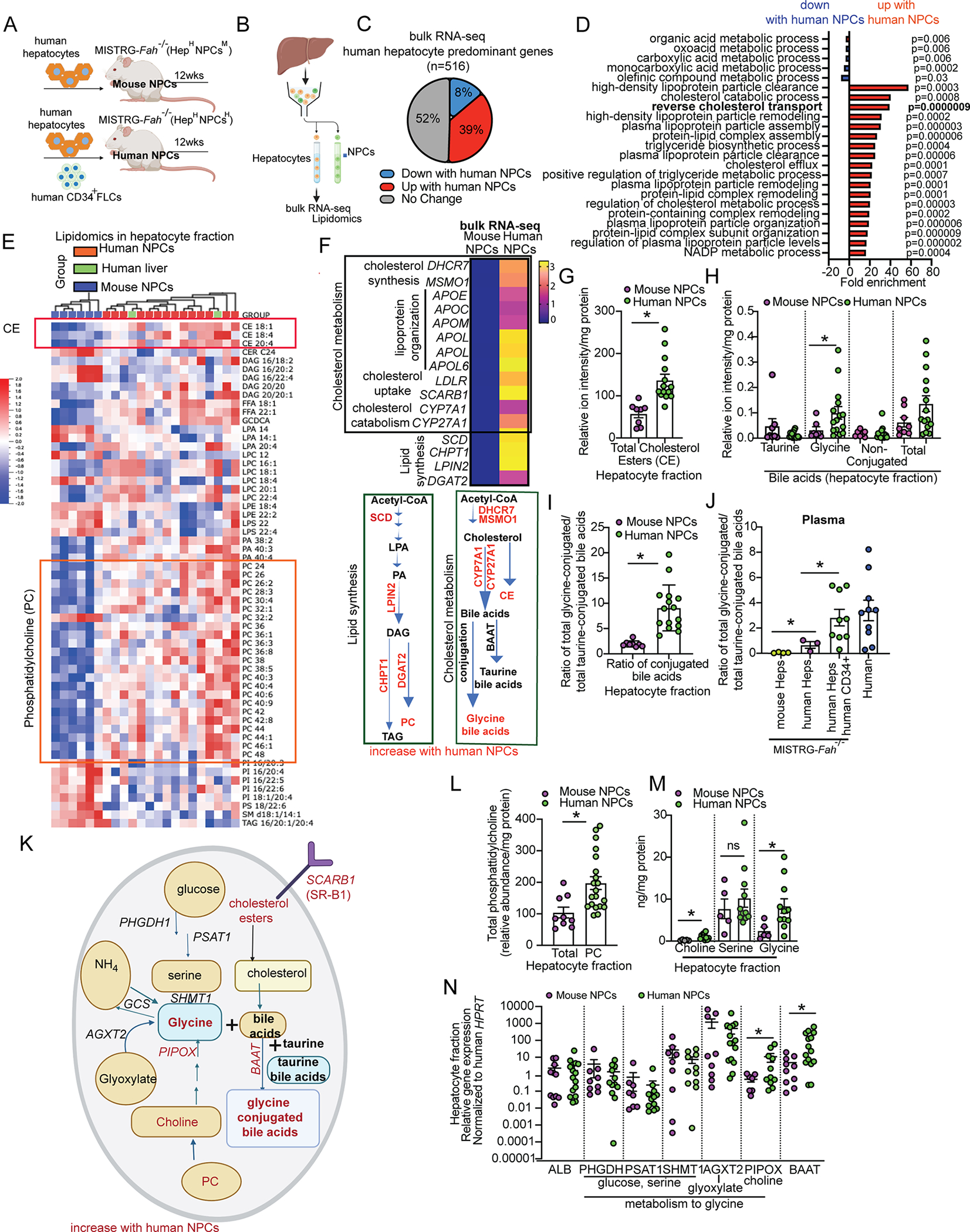
Human hepatocyte metabolic profile is shaped by NPCs **(A)** Adult MISTRG-*Fah*^−/−^ mice were engrafted with human hepatocytes and human CD34^+^ FLCs (human NPC group) or with only human hepatocytes (mouse NPC group). **(B)** Liver cells were fractionated into a hepatocyte-enriched fraction used for bulk RNA-seq analysis or lipidomics by HPLC-MS/MS. **(C)** Percentage of human-hepatocyte-predominant genes (n=516) that were altered in the presence of human NPCs. Gene expression levels were normalized to human albumin (*ALB*) expression (n=2 mice per group). **(D)** Gene Ontology (GO) enrichment analysis for biological functions in the differentially expressed genes. **(E)** Heatmap of significant lipids (p<0.03) measured by HPLC-MS/MS in the hepatocyte fraction after removal of mouse cells. Human hepatocytes isolated from healthy human liver tissue from liver donors served as a control. Hierarchical clustering was performed in the groups after log10 transformation of the lipid values using Qlucore Omics Explorer. Multi-group Kruskal-Wallis analysis was performed with selected variance having projection score Dim=3, filtered by standard variance <0.174. **(F)** Bulk RNA-seq in human hepatocytes: Average fold change in mice with human NPCs relative to mice with mouse NPCs. The equivalent lipid metabolism pathways are displayed. **(G-J)** HPLC-MS/MS analyses for cholesterol and bile acids in hepatocytes and in the plasma. For analyses in the plasma, MISTRG-*Fah*^−/−^ mice receiving mouse hepatocytes only and plasma from human healthy donors served as additional controls. **(K)** Schematic representation of the pathways of cholesterol and bile acid metabolism. Molecules that are upregulated in human hepatocytes in the presence of human NPCs are indicated in red. **(L-M)** Total phosphatidylcholine, choline, serine and glycine levels in the hepatocyte fraction, measured by HPLC-MS/MS. **(N)** Relative expression of human hepatocyte genes by RT-qPCR. Cartoon was made using BioRender. Each dot in the graphs is a biological replicate; n>3 biological replicates from 2 independent experiments are shown. Data represent mean ± SEM. *p < 0.05. See also Figure S6.

Human hepatocytes exert metabolic functions that are crucial for the whole body, however it remains unknown whether these are regulated in a cell-autonomous manner or by NPC derived signals. To address this question, we generated MISTRG-*Fah*^−/−^ mice engrafted with human hepatocytes and human CD34^+^ cell-derived NPCs (Hep^H^NPC^H^) or with human hepatocytes alone, thus bearing *murine* NPCs (Hep^H^NPC^M^) (Figure 4A). We hypothesized that the species-mismatch in the latter may reveal species-specific dependencies of human hepatocytes on NPCs for their metabolic functions. To understand the potential impact of NPC-derived signals on hepatocytes at the transcriptomic level, we performed RNA-seq analyses on hepatocytes isolated from MISTRG-*Fah*^−/−^ Hep^H^NPC^H^ and Hep^H^NPC^M^ mice (Figure 4B). Differential gene expression analyses, performed upon normalization to human albumin, showed that in the presence of human NPCs 39% of human hepatocyte-specific genes increased by ≥2-fold while 8% of the genes decreased by ≥2-fold (Figure 4C). Pathway analyses of the hepatocyte-specific genes strongly (> 10-fold) upregulated in the presence of human NPCs, revealed a significant enrichment of major metabolic pathways, including lipid metabolism, cholesterol metabolism and transport (Figure 4D).

On the basis of these findings, we characterized the lipidomic profile in hepatocytes of MISTRG-*Fah*^−/−^ Hep^H^NPC^H^ and Hep^H^NPC^M^ mice, by isolating hepatocytes and performing lipidomic analyses by high-performance liquid chromatography-tandem mass spectrometry (HPLC-MS/MS). These analyses demonstrated that when human NPCs are present, the lipidomic profile of human hepatocytes in a humanized liver is similar to that of the human liver (Figure 4E). However, in the absence of human NPCs, human hepatocytes displayed alterations in their lipidomic profile, deviating from the more human-like profile (Figure 4E). Lipids that significantly increased with human NPCs include cholesterol ester (CE) species, as well as phosphatidylcholine (PC), phosphatidic acid (PA) and free fatty acid (FFA) species as seen in human liver (Figure 4E). Of note, these differentially abundant lipids are in the same metabolic pathway as the components encoded by genes (*SCD, CHPT1*, *LPIN2*, *DGAT2, MSMO1*) that we found to be differentially expressed by RNA-seq (Figure 4F). This suggests that these lipid alterations in Hep^H^NPC^M^ mice stem from defects in biosynthesis rather than from defective transport.

### Bile acid metabolism in human hepatocytes is controlled by human NPCs

To further assess the role of human NPCs in controlling the metabolic function of human hepatocytes, we focused on cholesterol metabolism. The presence of human NPCs increased the expression of genes related to cholesterol biosynthesis (*MSMO1*, *DHCR7*), lipoprotein organization for cholesterol transport (*APOE*, *APOC1*, *APOM*, *APOL1*, *APOL2*, *APOL6*), cholesterol uptake (*SCARB1*, *LDLR*) and cholesterol catabolism to primary bile acids (*CYP7A1*, *CYP27A1*) (Figure 4F), along with the levels of total CE compared to control (Figure 4G). We confirmed that human NPCs increase the expression of a set of cholesterol pathway genes in hepatocytes also in the MISTRG6-RAF model, by qPCR in the hepatocyte fraction of mice showing a similar extent of humanization (Figure S6B). To understand the functional impact of these alterations, we focused on the metabolism of cholesterol to bile acids. Approximately 50% of circulating cholesterol is metabolized by the liver to bile acids^35^, which are then conjugated to glycine or taurine and secreted in the intestine where they emulsify dietary fats and lipid-soluble vitamins for absorption^36^. We generated MISTRG-*Fah*^−/−^ Hep^H^NPC^H^ and Hep^H^NPC^M^ mice and measured the abundance of bile acids in the liver and plasma by HPLC-MS/MS; mouse plasma from normal mice and human plasma samples from healthy donors served as a reference. These analyses showed that the levels of total non-conjugated bile acids in the hepatocyte fraction were not affected by human NPCs (Figure 4H). However, human NPCs shifted the conjugation of bile acids to the human-predominant glycine, leading to higher levels of glycine-conjugated bile acids and to an increased glycine/taurine conjugation ratio in the hepatocyte fraction (Figure 4H, I) and in the plasma, reaching levels similar to those of the human plasma (Figure 4J). These results show that bile acid conjugation in human hepatocytes is not a cell-autonomous mechanism and establish human NPCs as a regulator of the qualitative aspects of this metabolic process.

To understand how human NPCs control glycine conjugation to bile acids in human hepatocytes, we isolated human hepatocytes from MISTRG-*Fah*^−/−^ Hep^H^NPC^H^ and Hep^H^NPC^M^ mice and measured 1) the abundance of glycine precursor molecules (phosphatidyl-choline, choline and serine) by HPLC-MS/MS analyses and 2) the expression of all genes involved in glycine synthesis and conjugation to bile acids (Figure 4K). We found that the levels of phosphatidyl-choline (Figure 4L), choline and glycine (Figure 4M) were strongly induced in human hepatocytes in the presence of human NPCs. We also found that human NPCs 1) induce in human hepatocytes the expression of *BAAT*, the enzyme that mediates glycine or taurine conjugation to bile acids, 2) regulate genes involved in glycine synthesis from choline (*PIPOX*) (Figure 4N) and 3) regulate genes involved in the synthesis of phosphatidyl-choline, which is the major source of endogenous choline^37^ for the liver (Figure 4F, Figure S6B). These results establish human NPCs as a major paracrine regulator of bile acid glycine conjugation in human hepatocytes.

To corroborate these results, we also generated Hep^H^NPC^H^ mice with the MISTRG6-RAF approach, along with controls engrafted with human hepatocytes alone (Hep^H^NPC^M^) or human hepatocytes and mouse FLCs (Hep^H^NPC^M+MFL^) (Figure S6A). These mouse FLCs include the mouse hematopoietic stem cells that can support all the immune cell lineages in the MISTRG6 mice unlike the Hep^H^NPC^M^ mice that lack mature mouse T and B lymphocytes as a result of *Rag* deficiency. We measured CE, phosphatidylcholine, choline, serine and glycine levels in isolated hepatocytes by HPLC-MS/MS. With these experiments we ascertained that the lack of mouse lymphoid cells in Hep^H^NPC^M^ mice has no impact on human hepatocyte metabolism. Specifically, we compared Hep^H^NPC^M^ mice to the Hep^H^NPC^M+MFL^ configuration, which have functional mouse lymphoid cells. Moreover, and in agreement with our findings in the MISTRG-*Fah*^−/−^ system, we found that CE, phosphatidylcholine, choline and glycine levels as well as the ratio of glycine to taurine-conjugated bile acids were all increased by the presence of human NPCs (Figure S6C–I). Altogether, our results from two independent systems of liver humanization provide proof-of-concept that key metabolic functions of human hepatocytes are orchestrated by NPCs.

### WNT2 is a paracrine regulator of human hepatocyte metabolism.

**Figure 5.**
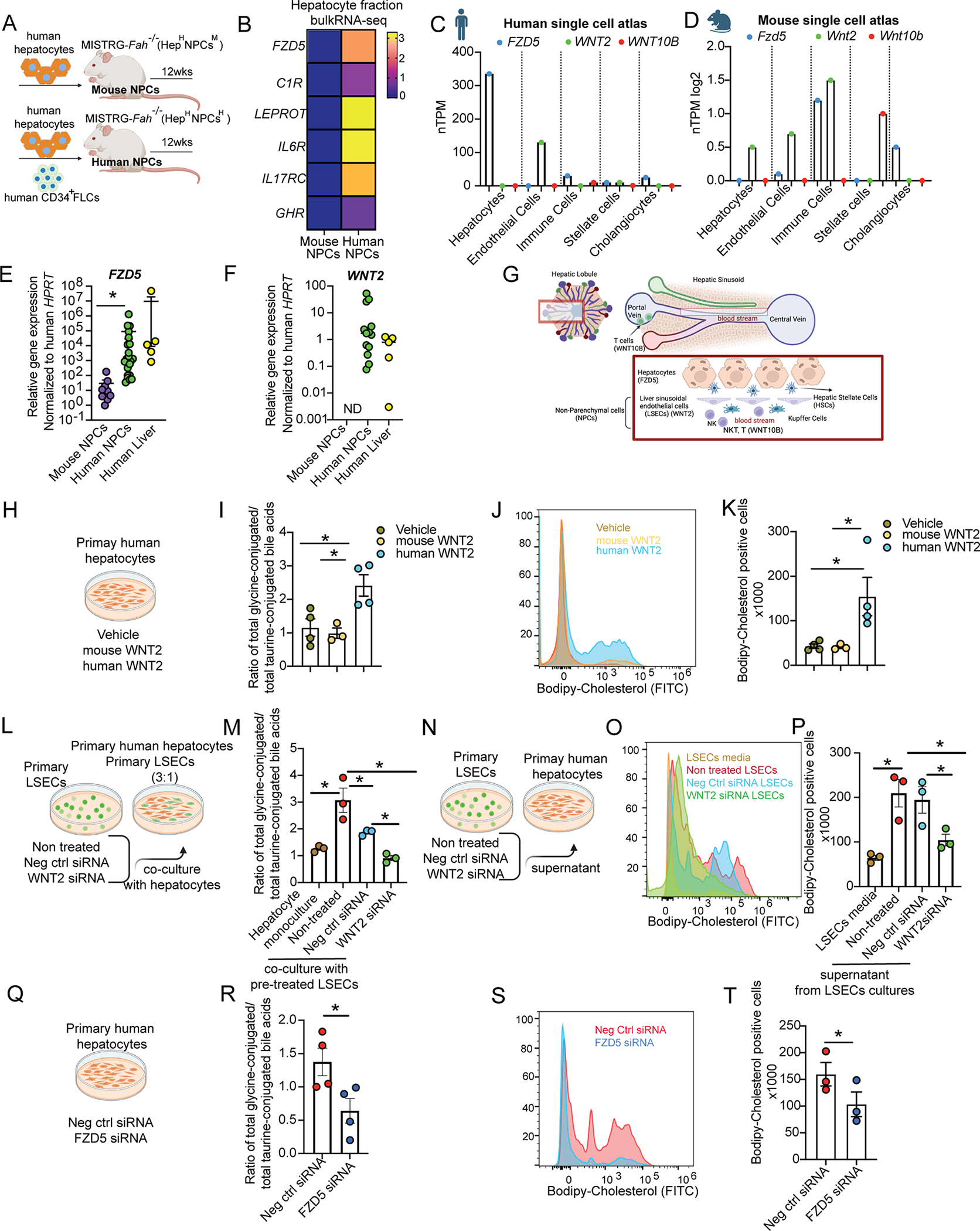
Endothelial cell-derived WNT2 regulates the metabolic function of human hepatocytes through FZD5. **(A)** Adult MISTRG-*Fah*^−/−^ mice were engrafted with human hepatocytes and human CD34^+^ FLCs (human NPC group) or with only human hepatocytes (mouse NPC group). **(B)** Bulk RNA-seq in human hepatocytes. Average fold change in the expression of receptor genes in mice with human NPCs relative to mice with mouse NPCs. **(C,D)** Gene expression of human/mouse *FZD5* and their ligands across different cell types in human and mouse liver atlas datasets^1^. See also Figure S7. **(E, F)** Human *FZD5* and WNT2 gene expression by RT-qPCR in the hepatocyte fraction from MISTRG-*Fah*^−/−^ mice and in healthy human liver tissue from partial hepatectomies. **(G)** Schematic representation of liver cell location. Cartoon was made using BioRender. **(H-K)** Primary human hepatocytes treated with human WNT2, mouse Wnt2 or vehicle (DMSO) for 24 hours. **(L-P)** Co-culture for 24 hours of primary human hepatocytes with primary human LSESs directly in the same plate or indirectly after supernatant transfer from LSECs. Primary human LSECs were pre-treated before co-culture for 24hours with negative control (Neg ctrl) siRNA or *WNT2* siRNA or left untreated. **(Q-T)** Primary human hepatocytes treated with siRNA silencing human *FZD5* or its negative control (neg ctrl) for 24 hours. **(H-T)** Primary bile acids were measured by HPLC-MS/MS in hepatocytes. Hepatocytes were treated with labeled cholesterol (Bodipy) for two hours and analyzed by flow cytometry. Each dot in the graphs is a biological replicate from at least 2 independent experiments. Data represent mean ± SEM. *p <0.05. See also Figure S8.

To identify paracrine signals through which human NPCs control the metabolic function of human hepatocytes, we examined which receptors are expressed in human hepatocytes and are further induced by human NPCs. We identified *FZD5*, *IL6R, C1R, LEPROT, IL17RC* and *GHR* as such (Figure 5A, B). Of these genes, we noted that *FZD5* (frizzled class receptor 5), a receptor of WNT ligands, is specifically expressed in human (Figure S7A, Figure 5C) but not in mouse hepatocytes^38^ (Figure S7E, Figure 5D). This prompted us to further study its role in the species-specific functions of the humanized liver. We validated that human NPCs induce FZD5 expression in hepatocytes in a larger cohort of humanized mice (Figure 5E). We observed that two established ligands of FZD5, WNT2 and WNT10B, are not expressed by hepatocytes but rather by endothelial cells (*WNT2*), stellate cells (*WNT2*, *WNT10B*) and T-cells (*WNT10B*) (Figure S7B, D), and therefore represent candidate paracrine regulators of human hepatocyte function. We focused on WNT2 as it displays a higher expression than WNT10B in the human liver (Figure S7B, D, Figure 5C) and is abundantly expressed by LSECs in the human liver (Figure S7B, Figure 5C), whereas in the mouse liver it is expressed by all liver cell types, including hepatocytes (Figure S7E, Figure 5D). Indeed, we found that the *WNT2* gene is also expressed in humanized livers at levels comparable to human liver (Figure 5F).

To assess the human specificity of hepatocyte regulation by WNT2, we treated human hepatocytes *ex vivo* with mouse or human WNT2 (Figure S8A). Mass spectrometry analyses showed that human but not mouse WNT2 increased CE levels in human hepatocytes (Figure S8B) and its metabolism to the primary bile acids CDCA through increased expression of CYP7A1 (Figure S8C, D). Moreover, human but not mouse WNT2 increased the levels of glycine-conjugated bile acids (Figure 5H, I Figure S8E), phosphatidylcholine and glycine in human hepatocytes (Figure S8F, H). Human but not mouse WNT2 induced the expression of WNT-target genes (*AXIN2* and *FZD5*), as expected, but also of genes involved in cholesterol uptake (*SCARB1*) and glycine synthesis (*AGXT2*, *PIPOX*) (Figure S8I). Since SCARB1 is responsible for reverse cholesterol transport in the liver^39^, we directly assessed cholesterol transport in the hepatocyte (cholesterol uptake) by treating cells *in vitro* with fluorescent labeled cholesterol (bodipy-cholesterol) and measuring its intracellular levels by flow cytometry. We found that only human and not mouse WNT2 increases cholesterol uptake by human hepatocytes (Figure 5J, K). These results establish human WNT2 as a major regulator of human hepatocyte metabolism *in vitro*, which phenocopies the effect of human NPCs.

### Endothelial cell-derived WNT2 regulates the metabolic function of human hepatocytes through FZD5

We aimed to identify which cell type functions as the major paracrine regulator of human hepatocyte function via WNT2. We observed that LSECs are the primary site of *WNT2* gene expression in the human liver (Figure 5C, S7B) and LSECs closely interact with hepatocytes (Figure 5G). To study the paracrine interaction of human hepatocytes with LSECs, we cultured hepatocytes alone or in a co-culture with primary LSECs in which we silenced (or not) *WNT2* (Figure 5L). Mass spectrometry analyses showed that the ratio of glycine to taurine-conjugated bile acids of human hepatocytes was significantly increased by co-culture with the LSECs; however, silencing of *WNT2* in LSECs abrogated this effect (Figure 5M). We then obtained conditioned media from primary LSECs in which we silenced *WNT2* or control LSECs and treated primary human hepatocytes (Figure 5N). Flow cytometry analyses in hepatocytes showed that cholesterol uptake significantly increased with LSEC-conditioned media, however, silencing of *WNT2* in LSECs prevented this effect (Figure 5O, P). To identify the receptor for WNT2, we silenced *FZD5* in human hepatocytes and performed mass spectrometry analyses. Thus, we confirmed that this receptor mediates the effect of WNT2 on glycine production (Figure S8Q) from choline or glyoxylate (Figure S8P, R), glycine conjugation on bile acids (Figure 5Q, R), as well as *SCARB1* gene expression (Figure S8R) and cholesterol uptake (Figure 5S,T). However, FZD5 did not mediate the effect of WNT2 on cholesterol metabolism to CDCA (Figure S8L–M) and had a mild effect on CE (Figure S8K) and phosphatidylcholine levels (Figure S8O). In summary, both human liver endothelial-derived WNT2 and hepatocyte FZD5 control human hepatocyte cholesterol uptake, glycine production and its conjugation to bile acids.

**Figure 6.**
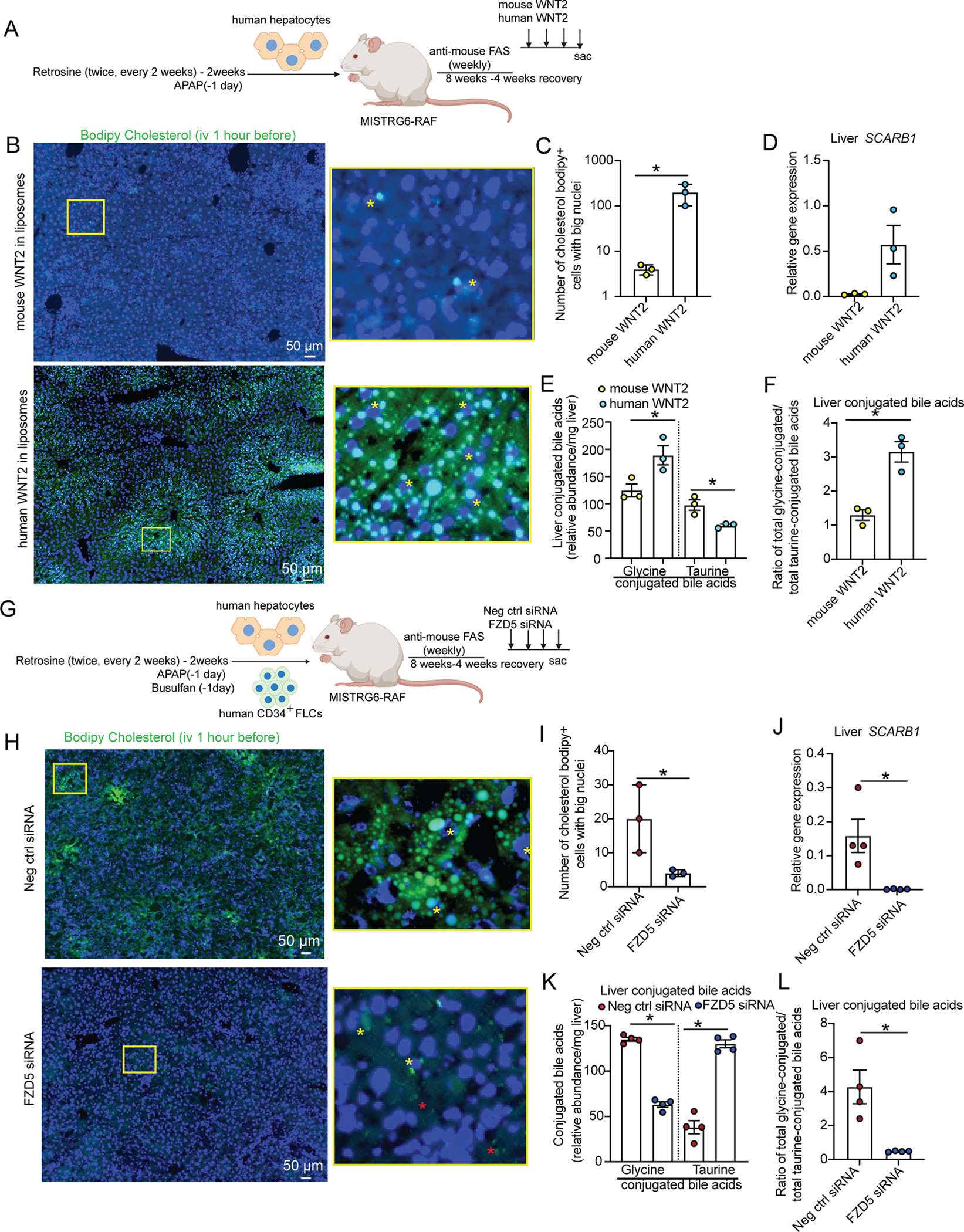
Human WNT2 and FZD5 are essential for liver cholesterol uptake and bile acid conjugation *in vivo.* **(A)** MISTRG6-RAF mice bearing human hepatocytes were daily treated with human or mouse WNT2 in liposomes, i.v. for 3 days. 24 hours after the last injection, liver and plasma were collected. **(B, C, H-I)** 1 hour before liver collection, mice were treated with bodipy-cholesterol i.v. Cells with big nuclei (indicative of hepatocytes) having green vesicles in the cytosol or close to the nucleus (indicated by yellow asterisk*) were counted. Small green vesicles (indicated by red asterisk) distant from big nuclei may indicate uptake from NPCs, therefore they were not counted. (Data are from 4 different fields per mouse at a 20x magnification). **(D, J)** Relative gene expression of *SCARB1* in the liver by RT-qPCR. **(E, F, K, L)** Bile acids in the liver measured by HPLC-MS/MS. **(G)** MISTRG6-RAF mice bearing human hepatocytes and human NPCs were daily treated with *FZD5* or Neg ctrl siRNAs through tail injections for 3 days. 24 hours after the last injection, liver and plasma were collected. Cartoon in 6A, 6G was made using BioRender. Each dot in the graphs is a biological replicate. Data represent mean ± SEM;*p <0.05.

Next, we examined the role of WNT2 and FZD5 in the regulation of human hepatocyte metabolism *in vivo*. For this purpose, we generated MISTRG6-RAF Hep^H^NPC^M^ mice, in which endothelial cells are murine, and injected them with liposomes loaded with human or mouse WNT2 (Figure 6A), based on previous *in vivo* studies with WNTs^40^. We found that human WNT2 in comparison to mouse WNT2 increased labeled cholesterol uptake by hepatocytes, determined by imaging (Figure 6B–C) and SCARB1 gene expression (Figure 6D), as well as the levels of glycine-conjugated bile acids (Figure 6E–F), whereas it decreased taurine-conjugated bile acids. To address the role of hepatocyte FZD5 in these processes *in vivo*, we generated MISTRG6-RAF Hep^H^NPC^H^ mice in which we ablated FZD5 in the liver through intravenous hydrodynamic delivery of FZD5 siRNAs (Figure 6G). We found that FZD5 ablation reduced cholesterol uptake by hepatocytes (Figure 6H,I) and *SCARB1* gene expression (Figure 6J) as well as the levels of glycine-conjugated bile acids whereas it increased taurine-conjugated bile acids (Figure 6 K, L). These results establish WNT2/FZD5 as a both necessary and sufficient paracrine mechanism through which endothelial cells regulate the metabolic function of human hepatocytes *in vivo*.

## DISCUSSION

The mechanisms that regulate the metabolic function of the liver have been unclear. In this study, 1) we developed a comprehensive and functional human hepatic tissue in a mouse host, achieving a cellular composition and a tissue architecture similar to that of the human liver, 2) we leveraged this technology to model common human liver diseases and also obtain proof-of-concept evidence that key metabolic functions of human hepatocytes are not cell-autonomous, but rather controlled by NPCs, 3) we revealed a human-specific paracrine mechanism whereby human endothelial WNT2 controls cholesterol uptake and bile acid conjugation in human hepatocytes through receptor FZD5. Our findings uncover the dependence of human hepatic metabolism on regulatory signals emanating from the local stromal microenvironment and reveal strong species-specificities in this context.

The comprehensive humanization of the MISTRG6 livers offers unique opportunities for the study of human liver biology and pathophysiology *in vivo*. We show that it is now feasible to study the interaction of human hepatocytes with their stromal microenvironment in a physiological context. With this system we can investigate the systemic impact of this crosstalk, such as the process of reverse cholesterol transport which is thought to be an important protective mechanism against atherosclerotic vascular disease^39^. Moreover, the successful development of human stellate cells in the humanized liver, and their activation in the context of a fibrotic response, is an important technological advance which will facilitate the prioritization and the functional assessment of therapeutic targets for liver fibrosis. In the same vein, the successful development of NAFLD in the humanized liver paves the way for the study of human-specific mechanisms that drive this prevalent liver disease, *in vivo*. Thus, MISTRG6 humanized liver-mice provide a valuable bridge between animal models and human studies, both for mechanistic understanding and therapeutic interrogation.

The control of cholesterol uptake and bile acid metabolism in human hepatocytes by LSECs, uncovers a hitherto unknown layer of regulation of human lipid metabolism. Endothelial WNT2 is required for liver zonation, liver repair and hepatocyte proliferation^18,41,42^, which suggests that endothelial regulation of lipid metabolism in hepatocytes is integrated with tissue morphogenesis, reflective of its vital importance.

The regulatory role of LSECs on the metabolic functions of hepatocytes may have significant implications in the context of disease, under conditions of impaired paracrine communication. Pericellular (chicken-wire) liver fibrosis represents one such condition, since activated stellate cells deposit excessive amounts of collagen in the space between LSECs and hepatocytes, thus creating a physical barrier that impedes their interaction. Thus, advanced liver fibrosis is associated with reduction of FZD5^43^ and an irregular bile acid profile with a decrease in glycine but not in taurine-conjugated bile acids^44^. These complications are relevant to the changes observed in our Hep^H^NPC^M^ humanized mice, in which hepatocytes lack proper regulation by LSECs.

Finally, our study establishes an important technological platform and an experimental approach to study cell-to-cell interactions in the human liver *in vivo*, in homeostasis and in diseases such as NAFLD and fibrosis, and a new tool to identify and evaluate therapeutic targets.

### Limitations of the study

We describe a chimeric humanized liver with high humanization of hepatocytes and immune cells (50–90%), partial humanization of LSECs and stellate cells (~50%) and very low humanization of cholangiocytes (1–10%) and portal fibroblasts. Neural cells, smooth muscle cells and endothelial cells in the blood vessels and in the lymphatic veins are still of mouse origin. Structurally, human cells may be located close to mouse cells which may influence their functional outcomes. Portal fibroblasts were not shown *in situ* due to the lack of good human-specific antibodies for IHC.

## STAR Methods

### RESOURCE AVAILABILITY

#### Lead Contact:

Further information and requests for resources and reagents should be directed to and will be fulfilled by the lead contact, Richard A Flavell (richard.flavell@yale.edu).

#### Materials Availability

The mouse lines described in the study are available under MTA from Regeneron Inc. Upon receipt of such MTA the mice will be made available by Yale University. Please contact Richard, A. Flavell richard.flavell@yale.edu and Donald Wiggin donald.wiggin@yale.edu

This study did not generate new unique reagents.

#### Data and Code availability

- Single-cell RNA-seq data have been deposited at GEO and are publicly available. Accession numbers are listed in the key resources table. This paper analyzes existing, publicly available data. These accession numbers are listed in the key resources table. All data reported in this paper will be shared by the lead contact upon request.
- This paper does not include any original code.
- Any additional information required to reanalyze the data reported in this paper is available from the lead contact upon request.

### EXPERIMENTAL MODEL AND STUDY PARTICIPANT DETAILS

#### Animals

MISTRG6 was generated by the Richard Flavell and Markus Manz laboratories and Regeneron Pharmaceuticals based on the *Rag2*^−/−^*IL2rg*^−/−^129xBalb/c background. In these mice, genes for human M-CSF (CSF1), IL3, SIRPα, thrombopoietin, GM-CSF (CSF2) and IL6 were knocked into their respective mouse loci ^15^. All human genes were brought to homozygosity except for the human SIRPα which was heterozygous to avoid phagocytosis of human cells from mouse macrophages. MIS^h/h^TRG6 were crossed with MITRG6 to generate MIS^h/m^TRG6 mice. MIS^h/h^TRG-*Fah*^−/−^ and MITRG-*Fah*^−/−^ were generated at Yale via the CRISPR/Cas9 system on a MISTRG or MITRG background ^16^. MIS^h/h^TRG-*Fah*^−/−^ were crossed with MITRG-*Fah*^−/−^ to generate MIS^h/m^TRG-*Fah*^−/−^ mice. Abbreviation of mouse strains throughout the manuscript is as follows: MIS^h/m^TRG-6 = MISTRG6, MIS^h/m^TRG-*Fah*^−/−^=MISTRG-*Fah*^−/−^. Both male and female mice were used in the study. Mice with Fah deletion were additionally maintained on 2-(2-nitro-4-trifluoromethylbenzoyl)-1,3-cyclohexanedione (NTBC, 1.6 μg/ml). All mice were maintained with cycling treatment with enrofloxacin in the drinking water (Baytril, 270 μg/ml) before engraftment with human cells. BALB/cJ mice (Jackson Laboratories) were used as donors of mouse hepatocytes and mouse fetal liver cells. All mice were maintained under specific pathogen-free conditions in our animal facilities (Biosafety Level (BSL) 2) under our Animal Studies Committee-approved protocol). Mice were housed on a 14-h light and 10-h dark cycle maintained at 40–60% humidity and at a temperature of 72°F ± 2°F. All animal experimentations were performed in compliance with the Yale Institutional Animal Care and Use Committee protocols. All mice used for the experiments were matched in the sex and were littermate controls. The age of mice used for experiments was between 12 and 18 weeks. Littermates of the same sex were randomly assigned to experimental groups. In all of our experiments both sexes were used and results are from mixed males and females.

#### Human participants

All human plasma samples were collected from healthy volunteers and provided by AlcHepNet. The donors did not have a history of liver-related disease or heavy alcohol consumption. All plasma donors were of a non-Hispanic origin and of white race. Informed consent was obtained from all subjects for blood sample collection as part of the AlcHepNet consortium observational study on acute alcohol associated hepatitis. Fresh human liver tissue was obtained from partial hepatectomies by the Yale Pathology Archives on the basis of Yale Human Investigation Committee protocols no. 0304025173, which allows retrieval of tissue from surgical pathology that was consented or has been approved for use with waiver of consent. Only tissue deemed healthy by a pathologist was used for experiments. The data were analyzed anonymously from preexisting patient databases and are thus exempt from consent by the human studies committee. Age, sex and other patient characteristics available are described in the Supplementary Table 1. We did not have access to information related to ancestry and socioeconomic status. Due to the limited number of human participants, we did not assess statistically the effect of sex in the analyses that we performed but the results provided in this study are from both sexes.

#### Primary cells

Primary human hepatocytes were either isolated from liver resections received as surgical waste (Supplementary Table 1 has the characteristics of the hepatocyte donor) or purchased from Thermo Fisher Scientific as cryopreserved human hepatocytes. Human hepatocytes were from three different donors (CellzDirect, Cat# HMCS1S HU8074: Caucasian, male, 68 years old; Cat# HMCS2S HU8093: African American, female, 31 years old; Cat# HMCS2S HU0965: Caucasian, female, 27 years old). All donors had no history of liver disease and alcohol consumption. The reason of death was stroke or N/A. Cryopreserved cells were placed in hepatocyte thaw media and after centrifugation at 200g, hepatocytes were diluted to HBSS. Morphology and viability were determined by Trypan blue exclusion in a hemocytometer Hepatocytes have been authenticated morphologically. Human primary liver endothelial cells were purchased from Cell biologics. Cells were tested negative for mycoplasma, bacteria, yeast, and fungi, HIV-1, hepatitis B and hepatitis C. The rest characteristics of the donor (age, sex or gender) are not available from the company. The LSECs have been authenticated by examining the protein expression of LSECs markers like CD31, LYVE1 by immunofluorescence.

### METHOD DETAILS

#### Transplantation of human CD34^+^ FLCs into mice

Fetal liver samples were cut into small fragments, treated for 45 min at 37 °C with collagenase D (Roche, 200 μg/ml) and prepared into a cell suspension. Human CD34^+^ cells were purified by performing density gradient centrifugation (Lymphocyte Separation Medium), followed by positive immunomagnetic selection with the EasySep Human CD34 Positive Selection Kit. For intra-hepatic engraftment, newborn 1–3-day-old pups were injected with 20,000 human fetal liver CD34^+^cells, or 100,000 CD34^−^ human fetal liver cells or their combination in 25 μl of PBS into the liver with a 29G needle. Adult mice, 6–8 weeks old, were injected with 100,000 human fetal liver CD34^+^ cells in 50 μl of PBS into the liver with a 29G needle after intraperitoneal administration of busulfan (30 mg/Kg) on the previous day. The use of all human materials was approved by the Yale University Human Investigation Committee.

#### Transplantation of human hepatocytes in mice

Adult MISTRG-*Fah*^−/−^ mice were transplanted as previously described^16^. In brief, NTBC water (1.6 μg/ml) was withdrawn 24 hours before transplantation. Six- to eight-week-old recipient mice were anesthetized under continuous inhalation of 5% (v/v) isoflurane in 1L/min oxygen. Cryopreserved human hepatocytes from three different donors were purchased from Thermo Fisher Scientific (CellzDirect, Cat# HMCS1S HU8074: Caucasian, male, 68 years old; Cat# HMCS2S HU8093: African American, female, 31 years old; Cat# HMCS2S HU0965: Caucasian, female, 27 years old). All donors had no history of liver disease and alcohol consumption. The reason of death was stroke or N/A. Frozen cell were placed in hepatocyte thaw media and after centrifugation at 200g, cells were diluted to HBSS. Cell number and viability were determined by Trypan blue exclusion in a hemocytometer. Mid-abdominal incisions were performed, and the lower pole of the spleen was injected with 1 million viable hepatocytes suspended in 50 μl of Dulbecco’s modified essential 73 medium (DMEM) via a 26G needle. The abdominal muscle layer and the skin were closed with 4–0 silk sutures (5 76 ETHILON^®^ Nylon Suture). Subsequently, NTBC water was completely withdrawn for 1 week and administered for 3 days when the mice appeared to be hypotonic or lost more than 10% of their weight. Transplantation of human CD34^+^ FLCs was performed on the same day with hepatocyte transplantation after one day from busulfan (30 mg/Kg) intraperitoneal injection. Mice were analyzed at least 12 weeks post-transplantation or sooner if moribund. In 5–6 weeks-old MISTRG6 mice, retrosine diluted initially in 100% ethanol at 20 mg/ml and then it was administered intraperitoneally at 60mg/Kg (15ul/gr mouse of 4mg/ml solution in 20% ethanol in PBS) twice at 2–3 weeks intervals, 2–3 weeks before human hepatocyte transplantation, as previously described^49^. One day before the intrasplenic human hepatocyte transplantation, the mice were treated with acetaminophen (APAP) (300 mg/Kg) intraperitoneally after 12h-16h of fasting. One week after human hepatocyte (same donors as in MISTRG-*Fah*^−/−^) transplantation and for 8 weeks, the MISTRG6 mice were injected weekly with anti-mouse FAS, 0.2mg/Kg (CD95-JO2) antibody (intraperitoneally diluted in PBS), as previously described ^50^. The mice were allowed to recover for 4 weeks after the last JO2 injection and then used for experiments.

#### Isolation of hepatocytes and NPCs from liver

Liver tissue resection from partial hepatectomies was collected from humans as surgical waste. Only tissue deemed healthy by the pathologist was used for experiments (RNA isolation, flow cytometry, HPLC-MS/MS analysis). The characteristics of the human hepatocyte donors are presented in supplementary Table 1. From humanized mice and from humans, liver tissue was collected in HBSS. Under aseptic conditions tissue was diced and washed in HBSS to remove excess blood. Briefly, tissue was minced on ice using two scalpels in a scissor motion. Tissue was diced until a slurry forms and tissue cannot be diced further (<3 mm). Tissue was transferred to a specimen container containing pre-warmed HBSS with 0.05% collagenase II, 0.5% fatty acid free BSA, 10mM CaCl_2_ and agitated (100 rpm) in a water bath with shaking bed for 30min, at 37 C. Cell suspensions were centrifuged twice (80g for 5 min, 4°C) to separate hepatocytes from NPCs.

#### Isolation of cells for flow cytometry

Single-cell suspensions were prepared from blood and liver. Mice were euthanized with 100% isoflurane. Blood was collected either retro-orbitally when the mouse was alive or via cardiac puncture after euthanasia. Livers were harvested, minced, and incubated in a digestion cocktail containing 0.5 mg/ml of collagenase II and 30 μg/ml of DNase I in HBSS at 37°C for 30 min. Tissue was then filtered through a 70-μm filter. The non-digested part that included the liver capsule and part of the biliary tree was further digested using Pronase-E 0.02% and combined with the initial digested part. Cells were treated with red blood cell lysis buffer and resuspended in PBS with 1% FBS. After centrifugation twice at 80 g to remove hepatocytes, the remaining NPCs were incubated at 4°C with anti-human and anti-mouse Fc block for 20 min. After washing, primary antibody staining was performed at 4°C for 20 min. After washing with PBS, cells were fixed using 4% paraformaldehyde. For intracellular staining, cells were washed with BD permeabilization buffer and stained in the same buffer for 45 min at room temperature. Samples were analyzed on an LSRII flow cytometer (BD Biosciences). Data were analyzed using FlowJo software version 3.2 and version 9.1.

#### Primary human hepatocyte culture and treatments

Primary human hepatocytes were either isolated from liver resections received as surgical waste (Supplementary Table 1 has the characteristics of the hepatocyte donor) or purchased from Thermo Fisher Scientific (CellzDirect, Cat# HMCS1S HU8074: Caucasian, male, 68 years old). Frozen cells were placed in hepatocyte thaw media and after centrifugation at 200g, cells were diluted to 1 million cells per ml in William’s E media containing supplements (2% human serum, 100nM dexamethasone, 100nM insulin and 0.375% fatty acid free BSA). Hepatocytes were looked in the microscope to validate their expected morphology and plated on type 1 collagen coated plates, at a density of 250,000/cm^2^. After cells had adhered (3–4h) media was removed and replaced with the same media supplemented with 5% FBS. The next day, hepatocytes were treated with recombinant human or mouse WNT2 (20 ng/ml) or its vehicle (DMSO) or were co-cultured with LSECs or silenced with siRNA for FZD5 (200nM) or for a universal negative control (200 nM). All treatments were performed for 24 hours.

#### Human primary LSECs culture and treatments

Human primary LSECs were used at passage 1–2 and kept in culture for only three days to reach confluency before co-culture with hepatocytes for another day. They were cultured in complete human endothelial cell medium. 1 day before co-culture with hepatocytes, they were silenced with WNT2 siRNA (200nM) or with a universal negative control siRNA (200nM) using Lipofectamine^™^ 2000 (Invitrogen).

#### WNT2 administration *in vivo*

Human recombinant WNT2 or mouse recombinant WNT2 was diluted in liposomes as previously described^40^. Briefly, 14 μmol of DMPC obtained from avanti polar lipids in chloroform was dried to a thin film in a 10 ml round bottom flask using nitrogen gas and was further evaporated in a vacuum overnight. Purified human or mouse Wnt2 in 1% CHAPS in 1× PBS was then diluted in 1× PBS to a total concentration of 1 μg/ml. This solution was then added to the 10 ml flask and vortexed vigorously until the solution was cloudy and there was no lipid visible on the bottom of the flask. 200 ul of this solution (200 ng/mouse) was injected in mice intravenously every day for 3 days. 20 hours after the last injection mice were euthanized.

#### *In vivo* FZD5 deletion

FZD5 MISSION siRNA (10 μg/mouse) was diluted in TransIT-QR hydrodynamic delivery solution and was daily injected through the tail vein for 3 days. 20 hours after the last injection the mice were euthanized. MISSION^®^ siRNA universal negative control (10 μg/mouse) was used as a control.

#### *In vivo* treatment with western diet

Adult MISTRG-*Fah*^−/−^ mice (6 weeks old) were engrafted with 100,000 human fetal liver CD34^+^cells and human hepatocytes. 12 weeks after human cell transplantation, mice were treated with Western diet (D18021203, Research diets) consisting of 40% fat, 40% sugars and 1% cholesterol. Dietary fat came mainly from partially hydrogenated corn oil. In addition, the drinking water was supplemented with high-fructose corn syrup (42 g/L) for 2 or 4 weeks. Control mice received chow diet for the same period.

#### *In vivo* treatment with CCl4

MISTRG6 pups (1–3-day-old) were injected with 20,000 human fetal liver CD34^+^cells or left non-engrafted. 12 weeks after human cell transplantation, mice were treated with 25% v/v carbon tetrachloride (CCl4) in corn oil or corn oil only (2.5 ul/gr of body weight) twice per week for 3 weeks. Mice were euthanized 24 hours after the last CCl4 injection.

#### *In vivo* treatment with DDC

MISTRG6 pups (1–3 days old) were injected with 20,000 human fetal liver CD34^+^cells or left non-engrafted. 12 weeks after human cell transplantation, mice were treated with DDC: 3,5-deithoxycarbonyl-1,4-dihydrocollidine 0.01% in their diet or normal chow diet ad libitum for 3 weeks.

#### *In vivo* treatment with Albumin-FITC

MISTRG6 pups (1–3 days old) were injected with 20,000 human fetal liver CD34^+^cells. 12 weeks after human cell transplantation, they were treated with FITC-Albumin (200 μg/mouse) intravenously in PBS 5 min before euthanasia. Livers were collected, incubated in 4% PFA for 4 hours and embedded in OCT media for cryosections.

#### BODIPY-Cholesterol *in vivo* and *in vitro*

Primary human hepatocytes were cultured in 6-well plates in Williams E medium with primary hepatocyte maintenance supplements. After treatments with siRNA for FZD5, WNT2 (mouse and human), supernatant from primary human LSECs, cells were incubated for 2 h with 2.5 μM BODIPY-cholesterol and rinsed three times. BODIPY fluorescence intensity of the cell pellet was measured with flow cytometry. Mice were injected intravenously with BODIPY Cholesterol (1 mg/Kg) 1 h before euthanasia. Livers were collected, incubated in 4% PFA for 4 hours and embedded in OCT media for cryosections and imaging in FITC channel in the microscope to see the green cholesterol droplet formation inside the cells.

#### Immunofluorescence-immunohistochemistry

Part of the liver tissue was incubated in 4 % PFA for 4 hours and then in 30% sucrose overnight and then stored in −80°C in OCT for cryosections and another part was put in 10% neutral buffered formalin, at 4°C, overnight and processed by paraffin embedding and sectioning by the Yale Pathology Tissue Services. Formalin-fixed, paraffin-embedded (FFPE) liver sections were deparaffinized in xylene (3 times for 10 min) and rehydrated through an ethanol gradient (100% to 50%). Antigen retrieval in FFPE sections was performed in a microwave for 30 min using 10 mM sodium citrate buffer, pH=6. Both FFPE and cryosections were blocked with 5% BSA for 1 hour and then incubated with primary antibodies or isotype controls diluted in 1% BSA at 4°C overnight. For intracellular epitopes, sections were incubated with 0.5% Tween-20 for 10 min before the primary antibodies. The next day sections were incubated for one hour with secondary antibodies diluted in 1% BSA. For immunofluorescence we used Alexa fluorochrome-conjugated secondary antibodies and the sections were mounted in mounting media with DAPI. For immunohistochemistry we used HRP-conjugated secondary antibodies after blocking the endogenous peroxidase with 3% H_2_O_2_. The sections were mounted in organic mounting media after incubation with the HRP-substate DAB for 10 min, counterstained with hematoxylin and dehydrated in xylene. For lipid staining we stained the sections for 15 min with LIPID-BODIPY 2 μM after incubation with the secondary Alexa antibodies.

#### Hematoxylin and Eosin staining

FFPE liver sections were deparaffinized in xylene (3 times for 10 min) and rehydrated through an ethanol gradient (100% to 50%). After hydration to distilled water, sections were immersed in hematoxylin for 1 min, rinsed in running tap water and differentiated in Scott’s tap water for 3 min. After rinsed in tap water, they were stained with Eosin-Y for 3 min and dehydrating in ethanol/xylene. The sections were mounted in DPX organic mounting media.

#### Sirius red staining

FFPE liver sections were deparaffinized in xylene (3 times for 10 min) and rehydrated through an ethanol gradient (100% to 50%). After hydration to distilled water, sections were immersed in Picro-Sirius red solution [(0.1% direct red 80 in saturated aqueous picric acid (1.2% picric acid in water)] for 60 min at room temperature, rinsed in absolute alcohol and dehydrated in 2 changes of absolute alcohol. The sections were mounted in DPX organic mounting media.

#### Trichrome staining

FFPE liver sections were deparaffinized in xylene (3 times for 10 min) and rehydrated through an ethanol gradient (100% to 50%). After hydration to distilled water, sections were fixed in Bouin’s solution at room temperature overnight. The next day was washed in running tap water to remove yellow color from sections. They were stained in working Gills Hematoxylin Solution for 5 minutes, washed in running tap water for 5 minutes and then rinsed in deionized water. Then, they stained in Biebrich Scarlet-Acid Fucshin for 5 minutes and rinsed in deionized water. Then they were placed in working Phosphotungstic/Phosphomolybdic Acid Solution for 5 minutes and then in Aniline Blue Solution for 20 minutes. Slides were rinsed in acetic acid 1%, for 2 minutes, rinsed in absolute alcohol and dehydrated in 2 changes of absolute alcohol. The sections were mounted in DPX organic mounting media.

#### Histological score

A board-certified pathologist of our group did a blinded semi-quantitative scoring analysis for the degree of ballooning, steatosis, ^51^ fibrosis, inflammation and ductular reaction using the standardized system ^52^ used in human specimens taking into account the degree and distribution of fibrosis. We also quantified the positive area of total collagen from Sirius red, aSMA, human pro-collagen 1 and human collagen 3 using Image J.

#### Gene expression analysis

RNA was extracted from whole liver tissue samples or isolated hepatocytes with the TRIzol reagent per manufacturer’s protocol. The High-Capacity cDNA Reverse Transcription Kit was used to make cDNA. RT–qPCR was performed using a SYBR FAST or TaqMan FAM universal qPCR kit. We used predesigned KiCqStart or TaqMan primers for *SCARB1, BAAT, ALB, FZD5, WNT2, AGXT2, PIPOX, PGHDH, PSAT1, CYP7A1, MSMO1, APOC1, LPIN2, CHPT1, AXIN2, HPRT1, COL1A1, Col1a1, Hprt1.* TaqMan primers are described in supplemental Table 2. Gene expression was normalized to human or mouse HPRT1. Individual values and means were plotted. The human specificity of the primers was examined by using mouse livers as negative control and human liver as positive control.

#### Bulk RNA sequencing

RNA isolated from homogenized liver tissue, or the hepatocyte fraction was used for transcriptome analysis. RNA was extracted from whole liver tissue samples or isolated hepatocytes with the TRIzol reagent per manufacturer’s protocol and then it was cleaned using the Qiagen mini-RNA kit per manufacturer’s protocol. Libraries were prepared at the Yale Center for Genomic Analysis and sequenced by NovaSeq. Raw sequencing reads were aligned to the human–mouse combined genome with STAR (https://doi.org/10.1093/bioinformatics/bts635), annotated and counted with HTSeq (https://doi.org/10.1093/bioinformatics/btu638), normalized using DESeq2 (https://doi.org/10.1186/s13059-014-0550-8) and graphed using the Broad Institute Morpheus web tool. Differential expression analysis was also performed with DESeq2. Corresponding pathway enrichment analysis of differentially expressed human genes was achieved using GeneOntology (http://geneontology.org/docs/go-enrichment-analysis/).

#### Single-cell RNA sequencing (10x Genomics)

Liver was harvested after overnight fasting and partial perfusion with PBS, minced, and incubated in a digestion cocktail containing 0.5 mg/ml of collagenase II (Sigma-Aldrich) and 30 μg/ml of DNase I (Sigma-Aldrich) in HBSS at 37°C for 30 min. The tissues were then filtered through a 70-μm filter. Cells were treated with red blood cell lysis buffer and resuspended in PBS with 1% FBS. After centrifugation once at 70 g to remove most hepatocytes, the remaining NPCs were cleaned from dead cell using the EasySep dead cell removal (annexin V) kit per manufacturer’s instructions and processed for droplet-based single-cell RNA sequencing. From this NPC-enriched fraction ~10,000 cells were encapsulated into droplets using the 10x Chromium GEM technology. Libraries were prepared in-house using Chromium Next GEM Single Cell 3′ Reagent Kit version 3.1 (10x Genomics). Single-cell RNA sequencing libraries were sequenced using NovaSeq. Raw sequencing reads were processed with Cell Ranger 3.1.0 using a human–mouse combined reference to generate a gene cell count matrix. To distinguish human and mouse cells, we counted the number of human genes (nHuman) and mouse genes (nMouse) with non-zero expression in each cell and selected cells with nHuman >20 × nMouse as human cells and cells with nMouse >10 × nHuman as mouse cells. Human Cells <20x nMouse were named mixed cells and were excluded from the analysis. The count matrix of human cells and human genes was used in the downstream analysis with Seurat 3.2^53^. Specifically, this matrix was filtered, retaining cells with more than 500 and fewer than 5,000 genes and more than 1000 UMIs. We then log-transformed each entry of the matrix by computing log (CPM/100 + 1), where CPM stands for counts per million. After normalization, we used adaptively thresholded low rank approximation (ALRA)^54^ to impute the matrix and fill in the technical dropped-out values. To visualize the cell subpopulations in two dimensions, we applied principal component analysis, followed by *t*-distributed stochastic neighbor embedding (*t*-SNE), a non-linear dimension reduction method for visualization. Louvain clustering was then used to generate clusters that were overlaid on the *t*-SNE embedding to investigate cell subpopulations. Marker genes for each cluster of cells were identified using the Wilcoxon test with Seurat.

A metagene score was assigned on the basis of publicly available single-cell RNA-seq datasets [human protein atlas, ^24,46^]. For each of these datasets we selected significantly differentially expressed genes from the 100 top genes and constructed a metagene defined as weighted average of the log-transformed expression of these differentially expressed genes with weights equal to the log fold ratio of these genes in the respective dataset. More specifically, if we assume we have a metagene M that contains m genes: $\left\{{gene}_{1},\;\;{gene}_{2},\ldots ,\;{gene}_{m}\right\}$ and each geneihas log fold change log ${FC}_{i}$ in the data we use for the signature of interest, and each geneihas an expression value of $x_{\text{gene}\;i}$ in a given cell in our dataset, then the score for M in this specific cell is calculated as:

$$S\left(M\right)=\sum_{i=1}^{m}X_{gene\text{−}i\;}\times \text{log}FC_{i}$$

Each cell from our single-cell dataset was characterized by a score associated with each of the metagenes.

#### Enzyme-linked immunosorbent assay (ELISA)

A human Albumin ELISA Kit was purchased from Bethyl Laboratories (Montgomery, TX, Cat# E88–129). Human Factor VIII ELISA Kit (E-EL-H6116) was purchased from Elabscience. Both ELISA assays were performed according to manufacturers’ instructions.

#### Depletion of mouse cells

For non-human specific lipids and metabolites, depletion of mouse cells was performed in the hepatocyte fraction prior to HPLC-MS/MS analysis. We depleted mouse cells using a mouse cell depletion kit from Miltenyi-Biotech according to manufacturers’ instructions.

#### Measurement of ALT

An ALT colorimetric assay kit was purchased from Cayman and was performed according to manufacturers’ instructions.

#### Measurement of lipoproteins

An assay kit was purchased from Fisher scientific (EHDL100) and was performed according to manufacturers’ instructions. The kit measuring VLDL, LDL as one factor and HDL as another factor. The ratio of both VLDL/LDL to HDL is the LDL/HDL in the Figure 3B.

#### HPLC-MS/MS analysis

EDTA plasma (100 μl) or homogenized liver tissue (~30 mg) or isolated hepatocytes were mixed with 900 μL ice-cold PBS spiked with an internal standard mixture in a glass tube. Then, 2 ml of chloroform and 1 ml of methanol were added in each sample. Samples, after vortexing for 1 min, were centrifuged at 4°C for 5 min at 2000 g. The lower organic phase (fraction A) containing the chloroform was collected with a Pasteur pipette. The upper phase containing methanol and saline was acidified to pH 3–4 with 10% formic acid. Samples were left on ice for 10 min and 1.5 ml of chloroform was added, followed by thorough vortexing for 1 min and centrifugation at 4°C for 5 min at 1500 g. The lower organic phase containing the chloroform was collected (fraction B) and neutralized to pH 6–7 with 5% ammonium hydroxide. Fractions A and B were evaporated to dryness and reconstituted in 200 μl of isopropanol:methanol:water (50:45:5). Lipids presented in supplemental Table 3 were measured in combined fraction A and fraction B. The upper methanol-water phase was evaporated to dryness and used for choline, glycine and serine measurement. All MS quantitation standards were purchased from Avanti Polar Lipids Inc. unless otherwise mentioned. All lipid and non-lipid species analyzed in this study were quantified using the multiple reaction monitoring (MRM) scanning method on an Agilent 6490-QQQ mass spectrometer. All data were acquired and analyzed using the Agilent Mass Hunter software. The LC separation was achieved as previously described ^55^ using a Gemini 5U C18 column (Phenomenex, 5 μm, 50 × 4.6 mm) coupled to a Gemini guard column (Phenomenex, 4 × 3 mm), using a gradient of buffer A, 95:5 (v/v) H2O/MeOH + 0.1% formic acid + 10 mM ammonium formate and buffer B, 60:35:5 (v/v) isopropanol (IPA)/MeOH/H2O + 0.1% (v/v) formic acid + 10 mM ammonium formate for the positive ionization and a gradient of buffer A, 95:5 (v/v) H2O/MeOH + 0.1% (v/v) NH4OH and buffer B, 60:35:5 (v/v) IPA/MeOH/H2O + 0.1% (v/v) NH4OH for the negative ionization. All the lipid estimations were performed using an electrospray ion (ESI) source, with following MS parameters: turbo spray ion source, medium collision gas, curtain gas = 20 L min^−1^, ion spray voltage = 4500 V (positive mode) or −5500 V (negative mode), at 400 °C. A typical LC-run was 55 min, with the following solvent run sequence post injection: 0.3 mL min–1 0% B for 5 min, 0.5 mL min–1 0% B for 5 min, 0.5 mL min–1 linear gradient of B from 0–100% over 25 min, 0.5 mL min–1 of 100% B for 10 min, and re-equilibration with 0.5 mL min–1 of 0% B for 10 min. A detailed list of all the species targeted in this MRM study, describing the precursor ion mass, the product ion targeted, and MS voltage parameters can be found in supplemental Table 3. All the lipid species were quantified by measuring the area under the curve in comparison to the respective internal standard, and then normalized to the internal standard ion intensity and to the total protein content of the liver tissue or cells (relative ion intensity). All the lipidomic data are represented as mean ± SEM of four (or more) biological replicates per group.

### QUANTIFICATION AND STATISTICAL ANALYSIS

Statistical analyses were performed with GraphPad Prism 9.01 unless otherwise is stated in the figure legends. Normality was tested and unpaired two-tailed Student’s *t*-test or two-tailed Mann-Whitney test was used to determine the statistical significance of a difference between two groups. *P*-values <0.05 were considered as statistically significant. Further details can be found in the figure legends.

## Supplementary Material

1 Figure S1. Related to Figure 1: Representative flow cytometry plots used for quantification of human liver immune and non-immune cells.
(A) Gating strategy for human and mouse immune cells of the blood and of the liver.
(B) Human liver immune cell subtypes after gating to human CD45.
(C) Human and mouse stellate cell and portal fibroblast-unique markers used for their quantification.
(D) Subtypes of LSECs based on their location in the hepatic lobule (zone 1 cells are close to the portal vein, zone 2 cells are between the portal and the central vein and zone 3 cells are close to the central vein). The anti-human antibodies were tested for their human specificity with mouse controls and after gating on human HLA-A,B,C positive, human CD45 negative cells.
(E) Gating strategy used to quantify human and mouse LSECs located in different zones. Since LSECs were shown to express CD45^45^, a common immune marker, we examined the expression of unique LSECs markers in the CD45 negative cells to avoid any contamination with immune cells.
(F) Gating strategy used to quantify human (human CK7^+^) and mouse (mouse EpCAM^+^) cholangiocytes. The gates for human antibodies were put based on non-engrafted mice.

2 Figure S2. Related to Figure 1: Human CD34^+^ FLCs support the same degree of NPC humanization in several models.
(A) Liver humanization models employed, with or without liver damage. MISTRG6 mice were engrafted at a neonatal age with human CD34^+^ FLCs. These mice have mouse hepatocytes and human NPCs but no liver damage. Adult MISTRG-*Fah*^−/−^ mice were treated with busulfan and on the next day were engrafted with human CD34^+^FLCs and mouse hepatocytes isolated from BALB/cJ donors or with human adult hepatocytes. NTBC was discontinued for 14 days to induce mouse hepatocyte death. Adult MISTRG6 mice were engrafted with human CD34^+^ FLCs and human adult hepatocytes after treatment with retrosine (two doses with a two-week interval), acetaminophen (APAP) and busulfan one day before human cell transplantation. One week after human hepatocyte engraftment, mice were treated with anti-mouse FAS weekly for 8 weeks to induce apoptosis in mouse hepatocytes only. These mice were named MISTRG6-RAF after the initials of the treatments used to help hepatocyte humanization (R: Retrosine, A: APAP, F: anti-FAS,). Cartoon was made using BioRender.
(B-D) Percentage of humanization for B) liver immune cells, C) liver LSECs (VAP1^+^, CD31^+^, LYVE1^+^) and D) stellate cells (Desmin^+^ and GFAP^+^).
(E) Percentage of human immune cells in the blood at different time points after human cell transplantation in *MISTRG-Fah*^−/−^ mice.
(F) Percentage of human immune cells in the blood.
(G) Human plasma albumin measured with ELISA 12 weeks after human cell transplantation.
(H) Immunofluorescence for human Hep Par1 in the liver of non-engrafted mice (mouse), in the healthy human liver, in MISTRG-*Fah*^−/−^ and in MISTRG6-RAF mice engrafted with human hepatocytes and human CD34^+^ FLCs, 12 weeks after human cell transplantation. A whole lobe of the liver was scanned with a Keyence microscope.
Mice were used at 12 weeks post-transplantation. Each dot in the graphs represents a biological replicate; n>4 biological replicates from 3 independent experiments. All mice within the same experiment were engrafted with FLCs from the same donor. Data represent mean ± SEM. *p < 0.05.

3 **Figure S3. Related to** Figure 1: **Human immune cell transcriptional signatures in the humanized liver**.t-SNE plots displaying the expression of immune cell population metagenes and marker genes in the humanized liver single-cell dataset. Metagenes were calculated based upon public healthy human liver single-cell datasets^24^ and validated in other two datastes from human liver (human protein atlas^24,46^) for:
(A) NK cells (c1).
(B) αβ T-cells (c0, c20, c21, c18), c18 has several unique markers of MAIT cells.
(C) Non-conventional γδ T-cells (c15).
(D) B-cells (c5, c6), plasma cells (c7, c29).
(E) Macrophages (c3) and monocytes (c4) with a signature called “pro-inflammatory” according to a public available healthy human liver single-cell dataset ^24^ c3 has enriched expression of “inflammatory” chemokine/cytokines and inflammasome components (*CXCL2, CXCL8, CXCL9, CXCL10, CXCL11, CCL19, CCL20, IL1b, IL1a, NLRP3*). The pro-inflammatory macrophage signature marks also a small cluster (c24) that express hepatocyte unique genes (Figure S4A). The latter population could potentially be explained by doublets consisting of macrophages and hepatocytes, or hepatocytes being phagocytosed. c4 is enriched on genes that are found in human liver monocytes in a recent publicly available single-nuclear/single-cell spatial dataset from non-perfused human livers ^47^.
(F) Macrophages-Kupffer clusters (c8, c27) enriched in Kupffer cell genes according to ^47^ and mapped to the signature of the non-inflammatory/tolerogenic or immuno-modulatory liver macrophages (non-inflammatory macrophages according to^24^) (c8, c27).
(G) Dendritic cells (DC) (c19). We didn’t have a signature from a healthy human liver to create a metagene but this cluster is enriched in conventional DC1 (cDC1) differentially expressed genes (DEGs) according to human liver single cell dataset ^47^ from patients with mixed pathologies (non-tumoural area of colorectal cancer liver metastasis, symptomatic cholecystolithiasis, obesity and Type 2 diabetes).

4 **Figure S4. Related to** Figure 1 **and** 2: **Human non-immune cell transcriptional signatures in the humanized liver**. t-SNE plots displaying the expression of non-immune cell population metagenes and marker genes in the humanized liver single-cell dataset. Metagenes were calculated based upon 3 different public human liver single-cell datasets (human protein atlas^24,46^).
(A) Human hepatocyte and zone-specific hepatocyte genes (zone 1 metagene and specific gene: c24 and half of c10, zone 2: c14, zone 3: c9). c24 is also expressing macrophage markers (Figure S3E) and possibly this cluster is doublets consisting of macrophages and hepatocytes, or hepatocytes being phagocytosed. c2 is expressing hepatocyte markers and markers of hepatocyte progenitors (CD24, SOX4).
(B) Human cholangiocyte metagene and markers (half of c10).
(C) Human hepatic stellate cell metagene and markers. Stellate cells are in two different clusters. One cluster expressing *LRAT*, which marks quiescent stellate cells^48^ (c11), and one cluster expressing *COL3A1*, *LOX* which marks activated stellate cells^48^ (c23). *COL3A1, LOX, COL6A1* are also expressed by portal fibroblasts. *ELN* is expressed by portal fibroblasts and not by stellate cells in a normal liver only. Thus, c23 may include both activated stellate cells and portal fibroblasts.
(D) Zone 2,3 LSECs (c17) are *CLEC1B-high*, *VWF-low* and co-express markers with macrophages (*PECAM1*, *CXCL16*, *CD36*).
(E) Pericentral LSEC metagene and markers expressed by this zone (c22).
(F) Zone 1 or periportal LSEC metagene and markers expressed by this zone (c13).
(G) Venous endothelial cell metagene marks most LSEC clusters (c22, c12, c13) as in human liver LSECs (human protein atlas^24,46^) but is not enriched in any cluster indicating possibly the absence of human venous endothelial cells in our dataset.
(H) Markers of LSECs not specific for a zone. Predominant venous endothelial gene (PTGDS) and LSECs genes are expressed mainly on c12. c11 (stellate cell cluster) has several genes in common with c12 as in the human liver single cell datasets (human protein atlas^24,46^).

5 Figure S5. Related to Figure 2 and Figure 3: Immunohistochemical analysis.
(A) Immunohistochemistry for CD34 (human and mouse, dual specificity), a marker of fetal/immature LSECs and venous endothelial cells. A negative area (N), including sinusoids (S) and the surrounding hepatocytes (H) is shown at a higher magnification. The lack of CD34 in sinusoidal endothelial cells located between hepatocytes indicates their maturity.
(B) Venous endothelial cells (black arrows) are positive for CD34, as expected in the adult liver.
(C) Immunohistochemistry for human CD68, a macrophage marker. Human macrophages are detected in all three zones of the liver (zone 1, zone 2, zone 3). The lack of human CD68 staining in the liver of mice non-engrafted with human cells confirms the human specificity of the antibody. Human CD68^+^ cells are located between hepatocytes (cells with big nuclei, indicated with H) in the sinusoids (indicated with S). All livers were collected from MISTRG6-RAF mice 12 weeks after human CD34^+^ FLC and human hepatocyte transplantation.
(D) H&E showing A) Mallory bodies (red arrow) ballooning (yellow arrow) and B) steatotic hepatocytes enriched in zone 3 but not in zone 1 that is the area close to porta vein (PV).
(E) Immunofluorescence for human CD68 (in red) and staining with BODIPY for lipids (in green) and counterstaining with DAPI in blue.
(F) IHC for human Cyp2E1(zone 2–3 hepatocyte marker) shows human steatotic hepatocytes in the zone 3 area.
(G) H&E and trichrome staining in engrafted and non-engrafted MISTRG6 mice treated with DDC.
(H) Histological score for ductular reaction, portal inflammation and portal fibrosis in engrafted and non-engrafted MISTRG6 mice treated with DDC.
(I) IHC for human collagen-3 (in purple) and aSMA (in green) in mice treated with CCl4 or DDC. The lack of human collagen-3 staining in the liver of mice that were not engrafted with human cells confirms the human specificity of the antibody.

6 Figure S6. Related to Figure 4: Human NPCs shape hepatocyte metabolism in MISTRG6-RAF mice.
(A) Adult MISTRG6-RAF mice were engrafted with human hepatocytes only or with human hepatocytes with mouse FLCs (gestational age 15 days) or with human CD34^+^ FLCs after treatment with retrosine and APAP. The mouse or human FLC group also received busulfan 1 day before transplantation. Following anti-mouse FAS antibody treatment weekly for 8 weeks, the mice were euthanized 4 weeks after the last anti-FAS treatment. Cartoon was made using BioRender.
(B) RT-qPCR in the hepatocyte fraction of MISTRG-RAF mice receiving human hepatocytes alone (mouse NPC group) or together with human CD34^+^ FLCs (human NPC group). We examined genes involved in cholesterol synthesis (*MSMO1*), uptake (*SCARB1*), transport (*APOC1*), catabolism to bile acids *(CYP7A1)* or PC synthesis (*CHPT1* and *LPIN2*).
(C-I) Metabolites measured by HPLC-MS/MS in all groups in the human hepatocyte fraction include: D) Total cholesterol esters (CE), E) Total phosphatidylcholine (PC), F) Choline, G) Serine, H) Glycine, I) ratio of total glycine to total taurine-conjugated bile acids.
Each dot in the graphs represents a biological replicate; n>3 biological replicates from 2 independent experiments. Data represent mean ± SEM. ns: non-significant, *p < 0.05.

7 Figure S7. Related to Figure 5: Species differences in the gene expression of *FZD5* and its ligands between human and mouse liver.
(A) *FZD5*, (B) *WNT2*, (C) *WNT5A*, (D) *WNT10B* gene expression in the human liver by single-cell RNA-seq from three different datasets (human protein atlas^24,46^). (E) *Fzd5* and *Wnt2* gene expression in the mouse liver by single-cell RNA-seq. Data retrieved from the mouse cell atlas.

8 Figure S8. Related to Figure 5: FZD5-dependent and FZD5-independent WNT2-mediated effects on human hepatocytes.
(A) Cultured primary human hepatocytes were treated with human or mouse WNT2 or its vehicle (DMSO) for 24 hours.
(B) Total cholesterol esters (CE) measured by HPLC-MS/MS.
(C) *CYP7A1* relative gene expression.
(D-H) HPLC-MS/MS analysis for D) primary non-conjugated bile acids (CA and CDCA), E) primary total glycine or taurine-conjugated bile acids, F) total phosphatidylcholine (PC), G) choline and H) glycine.
(I) Relative expression of genes involved in cholesterol uptake (*SCARB1*), PC synthesis (*LPIN2*), choline synthesis (*CHKA*), glycine synthesis (*AGXT2, PIPOX*), bile acid conjugation (*BAAT*) and of WNT2 target genes (*AXIN2*, *FZD5*) by RT-qPCR, displayed as fold-change to vehicle.
(J-R) Cultured primary human hepatocytes were transfected with FZD5 siRNA or negative control (Neg ctrl) for 24 hours. HPLC-MS/MS and RT-qPCR analyses were performed for: K) total cholesterol esters (CE), L) *Cyp7A1* relative gene expression, M) primary non-conjugated bile acids (CA and CDCA), N) primary total glycine or taurine-conjugated bile acids, O) total phosphatidylcholine (PC), P) choline, Q) glycine, R) genes involved in cholesterol uptake (*SCARB1*), PC synthesis (*LPIN2*), choline synthesis (*CHKA*), glycine synthesis (*AGXT2, PIPOX*), bile acid conjugation (*BAAT*) or WNT2 target genes, (*AXIN2, FZD5*). In K-R data are shown as fold change to vehicle.
(S) Schematic summary of FZD5-dependent and FZD5-independent effects of WNT2 on primary human hepatocytes.
Each dot in the graphs represents a biological replicate; Data represent mean ± SEM. *p < 0.05.

9 **Supplementary Table 1:** Human participant characteristics, related to Figure 1, 2, 3, 4 and 5

10 **Supplementary Table 2**: TaqMan and sigma primers related to figure s6, s8, 3, 5 and 6

11 **Supplementary Table 3**: MRM of lipids and metabolites related to figure 3, 4, 5, 6, s6 and s8

## Figures and Tables

**KEY RESOURCES TABLE:** 

REAGENT or RESOURCE	SOURCE	IDENTIFIER
**Antibodies**		
Rat anti-mouse CD45-BV605, clone, 30-F11	Bio Legend	Cat # 103139, RRID:AB_2562341
Rat anti-mouse CD45-A700, clone, 30-F11	Bio Legend	Cat # 103128, RRID:AB_493715
Rat anti-mouse CD45-PB, clone, 30-F11	Bio Legend	Cat # 103125, RRID:AB_493536
Mouse anti-mouse H-2Kb-PE-Cy7, clone: AF6-88.5	Bio Legend	Cat # 116520, RRID:AB_2721684
Rat anti-mouse CD31-FITC, clone MEC13.3	Bio Legend	Cat # 102514, RRID:AB_2161031
Rat anti-mouse CD31-PE, clone MEC13.3	Bio Legend	Cat # 102507, RRID:AB_312914
Rat anti-mouse Ep-CAM-APCCy7, clone G8.8	Bio Legend	Cat # 118218, RRID:AB_2098648
Rat anti-mouse Ep-CAM-APC, clone G8.8	Bio Legend	Cat # 118214, RRID:RAB_1134102
Mouse anti-human/mouse GFAP APC, clone 2E1.E9	Bio Legend	Cat # 644706, RRID:AB_2566110
Mouse anti-human/mouse GFAP FITC, clone 2E1.E9	Bio Legend	Cat # 644704, RRID:AB_2566109
Rat anti-mouse/human CD11b-FITC, clone M1/70	Bio Legend	Cat # 101206, RRID:AB_312789
Mouse anti-human B2m-FITC, clone 2M2	Bio Legend	Cat # 316304, RRID:AB_492837
Mouse anti-human CD31-PE, clone WM59	Bio Legend	Cat # 303106, RRID:AB_314332
Mouse anti-human CD31-APC, clone WM59	Bio Legend	Cat # 303115, RRID:AB_1877152
Mouse anti-human CD31-Alexa Fluor 594, clone WM59	Bio Legend	Cat # 303126, RRID:AB_2563303
Mouse anti-human CXCR6-APC, clone K041E5	Bio Legend	Cat # 356006, RRID:AB_2562223
Mouse anti-human CD90 (Thy1)-APC, clone 5E10	Bio Legend	Cat # 328114, RRID:AB_893431
Mouse anti-human CD56-PE-Cy7, clone MEM-188	Bio Legend	Cat # 304628, RRID:AB_2149542
Mouse anti-human CD45-PB, clone HI30	Bio Legend	Cat # 304029, RRID:AB_2174123
Mouse anti-human CD45-A700, clone HI30	Bio Legend	Cat # 304023, RRID:AB_493760
Mouse anti-human CD45-APC, clone HI30	Bio Legend	Cat # 304011, RRID:AB_314399
Mouse anti-human CD45-BV605, clone HI30	Bio Legend	Cat # 304042, RRID:AB_2562106
Mouse anti-human CD4-BV711, clone OKT4	Bio Legend	Cat # 317440, RRID:AB_2562912
Mouse anti-human CD45-BV605, clone HI30	Bio Legend	Cat # 304042, RRID:AB_2562106
Mouse anti-human CD8a-BV421, clone HIT8a	Bio Legend	Cat # 300928,RRID:AB_10612929
Mouse anti-human CD68-BV421, clone Y1/82A	Bio Legend	Cat # 333827, RRID:AB_2800881
Mouse anti-human CD36-APC-Cy7, clone 5–271	Bio Legend	Cat # 336213, RRID:AB_2072512
Mouse anti-human CD19-APC-Cy7, clone HIB19	Bio Legend	Cat # 302218, RRID:AB_314248
Rat anti-mouse CD90.2-PE, clone: 53–2.1	Bio Legend	Cat # 140308,RRID:AB_10641145
Mouse anti-human HLA-A,B,C-FITC, clone W6-32	Bio Legend	Cat # 311415, RRID:AB_493134
Mouse anti-human HLA-A,B,C-BV605, clone W6-32	Bio Legend	Cat # 311431, RRID:AB_2566150
Mouse anti-human LRAT, Clone: M34-P1F10	Novus Biologicals	Cat # NBP2-50444, RRID:N/A
Rabbit anti-human CD68	Invitrogen	Cat # PA5-83940, RRID:AB 2791092
Mouse anti-human CK7, clone KRT7/760	NSJ Bioreagents	Cat # V2658, RRID:N/A
Rabbit anti-human Collagen-3	BioRad	Cat # 2150-0100, RRID:AB_620309
Human anti-human Desmin, clone AbD03744	Bio-Rad	Cat # HCA023, RRID:AB_770095
Anti-human Desmin-FITC, clone REA1134	Miltenyi-Biotec	Cat #130-119-489, RRID:AB_2857460
Rabbit anti-mouse Desmin	Invitrogen	Cat # PA5-117909, RRID:AB_2902516
Rabbit anti-human MARCO	My BioSource	Cat # MBS9206180, RRID:N/A
Goat anti-mouse LYVE1	R&D	Cat # AF2125, RRID:AB_2297188
Rat anti-mouse LYVE1-PE, clone 223322	R&D	Cat # FAB2125P, RRID:AB_10889020
Goat anti-human LYVE1	R&D	Cat # AF2089, RRID:AB_355144
Mouse anti-human LYVE1-FITC	R&D	Cat # FAB20892G, RRID: N/A
Rabbit anti-human LYVE1	Abcam	Cat # Ab36993, RRID:AB_2138663
Rat anti-mouse VAP1, clone 7–88	Novus Biologicals	Cat # NBP1-58374,RRID:AB_11029742
Mouse anti-human VAP1-A700	R&D	Cat # IC39571N, RRID: N/A
Goat anti-human VAP1	Ray-biotech	Cat # 119-13209
Rat anti-human Pro-Collagen-1, clone M-58	Abcam	Cat # Ab64409, RRID:AB_1142324
Mouse anti-human/mouse aSMA-FITC, clone 1A4	Sigma Aldrich	Cat # F3777, RRID:AB_476977
Mouse anti-human PDGFRa-APC, clone 16A1	Bio Legend	Cat # 323511, RRID:AB_2783190
Rabbit anti-human FAH	LS Bio	Cat # LS-C482648/164400, RRID: N/A
Rabbit anti-human/mouse CD34, Clone SI16-01	Invitrogen	MA5-32059, RRID:AB_2809353
Rabbit anti-human Cyp2E1	NSJ Bioreagents	Cat # F51257, RRID: N/A
Mouse anti-human Hep Par1	Ray Biotech	Cat # 188-10224, RRID: N/A
Anti-mouse IgG-HRP-linked	Cell Signaling	Cat # 7076S, RRID: N/A
Anti-Rabbit IgG-HRP-linked	Cell Signaling	Cat# 7074S, RRID: N/A
Anti-Rat IgG-HRP-linked	Cell Signaling	Cat# 7077S, RRID: N/A
Anti-goat IgG-HRP-linked	Cell Signaling	Cat# 7074S, RRID: N/A
Goat anti-human IgG-Alexa Fluor 488 (H+L)	Invitrogen	Cat # A11013, RRID: N/A
Donkey anti-rabbit IgG-Alexa Fluor 488 (H+L)	Invitrogen	Cat # A21206, RRID:AB_2535792
Goat anti-mouse IgG-Alexa Fluor 488 (H+L)	Invitrogen	Cat # A11029, RRID:AB_2534088
Donkey anti-goat IgG-Alexa Fluor 488 (H+L)	Invitrogen	Cat # A11055, RRID: N/A
Donkey anti-rat IgG-Alexa Fluor 488 (H+L)	Invitrogen	Cat # A21208, RRID: N/A
Goat anti-rabbit IgG-Alexa Fluor 594 (H+L)	Invitrogen	Cat # A11072, RRID: N/A
Donkey anti-goat IgG-Alexa Fluor 555 (H+L)	Invitrogen	Cat # A21432, RRID: N/A
Donkey anti-rat IgG-Alexa Fluor 555 (H+L)	Invitrogen	Cat # A48270, RRID: N/A
Goat anti-mouse IgG-Alexa Fluor 594 (H+L)	Invitrogen	Cat # A11020, RRID: N/A
Donkey anti-goat IgG-Alexa Fluor 647 (H+L)	Invitrogen	Cat # A21447, RRID: N/A
Donkey anti-rabbit IgG-Alexa Fluor 647 (H+L)	Invitrogen	Cat # A32795, RRID: N/A
Donkey anti-mouse IgG-Alexa Fluor 647 (H+L)	Invitrogen	Cat # A32787, RRID: N/A
Goat anti-rat IgG-Alexa Fluor 647 (H+L)	Invitrogen	Cat # A21247, RRID: N/A
**Biological samples**		
Human fetal liver	Advanced Bioscience Resource Inc.	Cat# 9583
Primary human hepatocytes	Thermo Fisher Scientific	Cat# HMCS1S HU8074
Primary human hepatocytes	Thermo Fisher Scientific	Cat# HMCS2S HU8093
Primary human hepatocytes	Thermo Fisher Scientific	Cat# HMCS1S HU0965
		
**Chemicals, peptides, and recombinant proteins**		
NTBC (CuRx Nitisinone)	Yecuris	Cat# 20-0027
eBioscience^™^ Fixable Viability Dye eFluor^™^ 780	Thermo Fisher Scientific	Cat # 65-0865-14
eBioscience^™^ Fixable Viability Dye eFluor^™^ 506	Thermo Fisher Scientific	Cat # 65-0866-14
Ceramide/Sphingoid internal standard mixture I	Avanti Polar Lipids	Cat# LM6002
Glycocholic acid-d4	Avanti Polar Lipids	Cat# 330277
Glycochenodeoxycholic acid-d4	Avanti Polar Lipids	Cat# 330273W
Chenodeoxycholic acid-(2,2,4,4-d4)	Avanti Polar Lipids	Cat# 330259
Cholic acid-d4	Avanti Polar Lipids	Cat# 330256W
Yeast extract total	Avanti Polar Lipids	Cat# 1900000C
SPLASH^®^ LIPIDOMIX^®^ Mass Spec Standard II	Avanti Polar Lipids	Cat# 330709
Taurocholic acid (sodium salt)	Cayman Chemical	Cat# 16215
Taurohyodeoxycholic acid	Cayman Chemical	Cat# 21956
Ursodeoxycholic acid	Cayman Chemical	Cat# 15121
β-muricholic acid	Sigma-Aldrich	Cat# SML2372
α-muricholic acid	Cayman Chemical	Cat# 20291
Deoxycholic acid	Cambridge Isotope Laboratories, Inc.	Cat# ULM-9545
Lithocholic acid	Sigma-Aldrich	Cat# L6250
7-ketodeoxycholic acid	Sigma-Aldrich	Cat# SMB000806
7-ketolithocholic acid	Avanti Polar Lipids	Cat# 6708PIA010
Glycodeoxycholic acid	Sigma-Aldrich	Cat# 06863
Glycolithocholic acid	Cayman Chemical	Cat# 20273
Glycodeoxycholic acid, sodium salt	EMD, Millipore	Cat# 361311
Taurodeoxycholic acid, sodium salt hydrate	Cayman Chemical	Cat# 15935
Sodium taurolithocholate	Sigma-Aldrich	Cat# T7515
Glycocholic acid, sodium salt	EMD, Millipore	Cat# 360512
Taurochenodeoxycholic acid, sodium salt	Cambridge Isotope Laboratories, Inc.	Cat# ULM-9561
Sodium glycochenodeoxycholate	Sigma-Aldrich	Cat# G0759
Hyodeoxycholic acid	Sigma-Aldrich	Cat# H3878
Sodium chenodeoxycholate	Sigma-Aldrich	Cat# C8261
Cholic acid	Sigma-Aldrich	Cat# C1129
Choline Chloride	Sigma-Aldrich	Cat# C7017
Glycine	Sigma-Aldrich	Cat# 50046
L-Serine	Sigma-Aldrich	Cat# S4500
EDTA solution 0.5 M	Sigma-Aldrich	Cat# 324506
Chloroform for HPLC	Sigma-Aldrich	Cat# 650498
Chloroform for RNA	Sigma-Aldrich	Cat# C2432
BioReagent 2-Propanol, Molecular Biology Grade, Liquid, ≥99.5%	Sigma-Aldrich	Cat# I9516
Methanol LC-MS grade	Southern Labware	Cat# 1935-5
Isopropanol, Optima LC/MS grade	Fisher Scientific	Cat# A461500
mouse cell depletion kit	Miltenyi-Biotech	Cat# 130-104-694
Chromium Next GEM Single Cell 3_ Library Kit v3.1	10X Genomics	Cat# 1000157
Trizol	Invitrogen	Cat# 15596018
Rneasy Mini kit	Qiagen	Cat# 74106
iTaq Universal SYBR Green Supermix	Bio-Rad	Cat# 1725125
High-Capacity cDNA Reverse Transcription Kit	Thermo Fisher Scientific	Cat# 43-688-14
TaqMan^™^ Gene Expression Assay (FAM), XS	Thermo Fisher Scientific	Cat# 4448892
Mounting media with DAPI	Vector	Cat# H-1200-10
HRP-substate DAB	Vector	Cat# SK-4105
Ethanol 200%	Yale Medical Stockroom	Cat# N/A
Scott’s water	Sigma-Aldrich	Cat# S5134
Eosin Y Solution	Sigma-Aldrich	Cat# MKCL4995
Hematoxylin Solution	Sigma-Aldrich	Cat# SLCJ5092
DPX Mountant for histology, slide mounting media	Sigma-Aldrich	Cat# 06522
Hydrogen Peroxide 30% (W/W) Solution	Sigma-Aldrich	Cat# H1009
Xylene A.C.S Reagent	JT Baker	Cat# 9490-01
Tween-20	Sigma-Aldrich	Cat# P7949
Gill’s hematoxylin solution	Electron microscopy science	Cat# 26801-01
Eosin-Y solution w	Millipore-Sigma	Cat# 318906
Scott’s tap water substitute concentrate (10x)	Millipore-Sigma	Cat# S5134
Sodium Citrate Tri Basic Dihydrate	Sigma-Aldrich	Cat# S4641
Ethanol	Yale Medical Stockroom	Cat# N/A
Picric acid	Sigma	Cat# 197378
Direct Red 80	Sigma	Cat# 365548
10% neutral buffered formalin	Sigma-Aldrich	Cat# HT501128
DDC: 3,5-deithoxycarbonyl-1,4-dihydrocollidine	Millipore-Sigma	Cat# 137030
BODIPY-cholesterol	GLP BIO	Cat# GC42964
BODIPY-Lipids	GLP BIO	Cat# GC42959
Tissue-Tek O.C.T. Compound	VWR	Cat# 25608-930
FITC-Albumin	Millipore-Sigma	Cat# A9771
Corn Oil	Sigma-Aldrich	Cat# C8267
high-fructose corn syrup	Amazon	Cat# N/A
Carbon Tetrachloride, Anhydrous, >=99.5%	Sigma	Cat# 289116
Western diet	Research diets	Cat# D18021203
TransIT-QR hydrodynamic delivery solution	Mirus	Cat# MIR 5240
DMPC	Avanti Polar Lipids	Cat# 850345P-25mg
CHAPS	Life Technologies	Cat# 28300
recombinant human WNT2	Origene	Cat# TP762201
recombinant mouse WNT2	My BioSource	Cat# MBS957358
Baytril	Bayer Healthcare	Cat# 100-CA1
Collagenase D	Roche	Cat# 11088866001
Lymphocyte Separation Medium	Sigma-Aldrich	Cat# C-44010
EasySep Human CD34 Positive Selection Kit	STEMCELL Technologies	Cat# 17856
Busulfan	Sigma-Aldrich	Cat# B2635
Isoflurane	Covetrus	Cat# 029405
Trypan blue	Life Technologies	Cat# 15250061
HBSS	Life Technologies +	Cat# 14025092
DMEM	Thermo Fisher Scientific	Cat# 10565-018
Collagenase II	Gibco	Cat# 17101-015
AccuCheck and counting beads	Life technologies	Cat# PCB100
70-gm filter cell-strainer	Fisher Scientific	Cat# 22363548
Ultra Pure BSA	Life Technologies	Cat# AM2616
Pronase-E	Millipore	Cat# 53402
BD permeabilization buffer	y Fisher Scientific	Cat# BDB561651
DPBS	Gibco	Cat# 14190-144
16% Paraformaldehyde	EMS Acquisition	Cat# 15710
anti-human Fc blocker	BD Biosciences	Cat# 564220
anti-mouse Fc blocker	BD Biosciences	Cat# 553142
FBS	Sigma-Aldrich	Cat# F4135
red blood cell lysis buffer (10x)	BioLegend	Cat# 420302
EasySep Dead Cell Removal (Annexin V) Kit	Stemcell technologies	Cat# 17899
CACL2	Life Technologies	Cat# 509703
26G needle	BD	Cat# 305111
DNase I	Sigma-Aldrich	Cat# 10104159001
29G needle	Fisher Scientific	Cat# 1484132
4–0 silk sutures (5 76 ETHILON^®^ Nylon Suture)	Johnson and Johnson	Cat# 669G
Retrosine	Sigma-Aldrich	Cat# R0382
DMSO	Sigma-Aldrich	Cat# D2650
Acetaminophen (APAP)	Sigma-Aldrich	Cat# A5000
anti-mouse FAS, (CD95-JO2)	BD	Cat# BDB554254
MEM, NEAA	Life Technologies	Cat# 10370021
Gentamicin Sulfate	Sigma-Aldrich	Cat# G1264
Insulin solution from bovine pancreas	Sigma-Aldrich	Cat# I0516
CellAdhere^™^ Collaaen I-Coated, 6-Well Flat-Bottom Plate	Stem cell technologies	Cat# 100-0362
Dexamethasone 98% powder	Sigma-Aldrich	Cat# D1756
complete human endothelial cell medium	Cell biologics	Cat# H1168
Lipofectamine^™^ 2000	Life Technologies	Cat# L3000001
William’s E Medium, no phenol red	Gibco	Cat# A1217601
Primary Hepatocyte Maintenance Supplement	Gibco	Cat# CM4000
Hepatocyte Thaw Medium	Gibco	Cat# CM7500
DPX Mountant for histology	Millipore sigma	Cat# 44581
Bouin’s solution	Millipore sigma	Cat# HT1032
Gemini 5 pm C18 110 A, LC Column 50 × 3.0 mm	Phenomenex	Cat# Part No: 00B-4435-Y0-P
Security Guard Cartridges, Gemini C18 4 × 3.0mm- Holder Part No.	Phenomenex	Cat# Part No.: AJ0-7597
**Critical commercial assays**	**SOURCE**	**IDENTIFIER**
Human Albumin Elisa	Bethyl	Cat# E88-129
HDL, LDL/VLDL assay kit	EnzyChrom/ Fisher scientific	Cat# EHDL100
Human Factor VIII ELISA Kit	Elabscience	Cat# E-EL-H6116
Trichrome Stain (Masson) Kit	Sigma Aldrich	Cat# HT15-1KT
Mouse cell depletion kit	Miltenyi-Biotech	Cat#130-104-694
EasySep Dead cell removal (annexin V) kit	Stem cell technologies	Cat#17899
ALT assay kit	Cayman	Cat# 700260
Deposited data	SOURCE	IDENTIFIER
Bulk RNA sequencing analysis of the humanized liver tissue	This paper	Cat# GSE234755
Bulk RNA sequencing analysis of hepatocytes isolated from humanized liver	This paper	Cat# GSE234757
Single-cell RNA sequencing analysis of humanized liver	This paper	Cat# GSE234758
Humanized Liver	This paper	Cat# GSE234759
**Experimental models: Cell lines**	**SOURCE**	**IDENTIFIER**
Human primary liver sinusoidal endothelial cells	Cell biologics	Cat# H-6017
**Experimental models: Organisms/strains**	**SOURCE**	**IDENTIFIER**
MITRG-*Fah−/−*	Flavell lab	Cat# N/A
MIS^h/h^TRG-*Fah−/−*	Flavell lab	Cat# N/A
MIS^h/m^TRG-*Fah−/−*	Flavell lab	Cat# N/A
BALB/cJ	Jackson	Cat# 000651
MIS^h/h^TRG6	Flavell lab	Cat# N/A
MITRG6	Flavell lab	Cat# N/A
MIS^h/m^TRG6	Flavell lab	Cat# N/A
MIS^h/h^TRG-*Fah−/−*	Flavell Lab	Cat# N/A
**Oligonucleotides**	**SOURCE**	**IDENTIFIER**
MISSION^®^ siRNA Universal Negative Control	Thermo-Fisher	Cat # SIC001 and SIC002
siRNA for WNT2	Thermo-Fisher	Cat # 4392420
MISSION siRNA for FZD5	Thermo-Fisher	Cat # EHU125691
See Table S1 for a list of oligonucleotides	This paper	
**Software and algorithms**	**SOURCE**	**IDENTIFIER**
Qlucore Omics Explorer version 3.9	Yale software library	https://qlucore.com/omics-explorer
Image J	NIH page	https://imagei.nih.gov/ii/download.html
FlowJo software version 3.2 and version 9.1.	FlowJo	https://www.flowio.com
GraphPad, Prism version 8 and 9	Yale software library	https://www.graphpad.com
Mass Hunter Qualitative analysis	Agilent Technologies, Inc.	https://www.agilent.com/en/product/software-informatics/mass-spectrometry-software/data-analysis/qualitative-analysis
Biorender	Biorender	https://www.biorender.com
**Other** **Public available databases used in this paper**	**SOURCE**	**Link to the dataset**
Human protein atlas-single cell data in the liver	Pubmed	https://www.proteinatlas.org
MacParland et al, Nature Communications 2018	Pubmed	http://shiny.baderlab.org/HumanLiverAtlas/HumanLiver/
Aizarani et al, Nature 2019	Pubmed	http://human-liver-cell-atlas.ie-freiburg.mpg.de
Guilliams et al, Cell 2022	Pubmed	https://www.livercellatlas.org/umap-humanAll.php

## Highlights

- Development of a comprehensive and functional human hepatic tissue in a mouse host
- Modeling NAFLD and fibrosis in human cells *in vivo*
- Key metabolic functions of human hepatocytes are controlled by NPCs
- WNT2 controls cholesterol uptake and bile acid conjugation in hepatocytes through FZD5.

## Inclusion and Diversity

We worked to ensure gender balance in the recruitment of human subjects. We worked to ensure sex balance in the selection of non-human subjects. We support inclusive, diverse, and equitable conduct of research.
