# Supplementary figures and images for: Humanized mouse liver reveals endothelial control of essential hepatic metabolic functions
Source: Cell. Author manuscript; Available in PMC 2024 Aug 31. (PMC10544749; doi:10.1016/j.cell.2023.07.017)

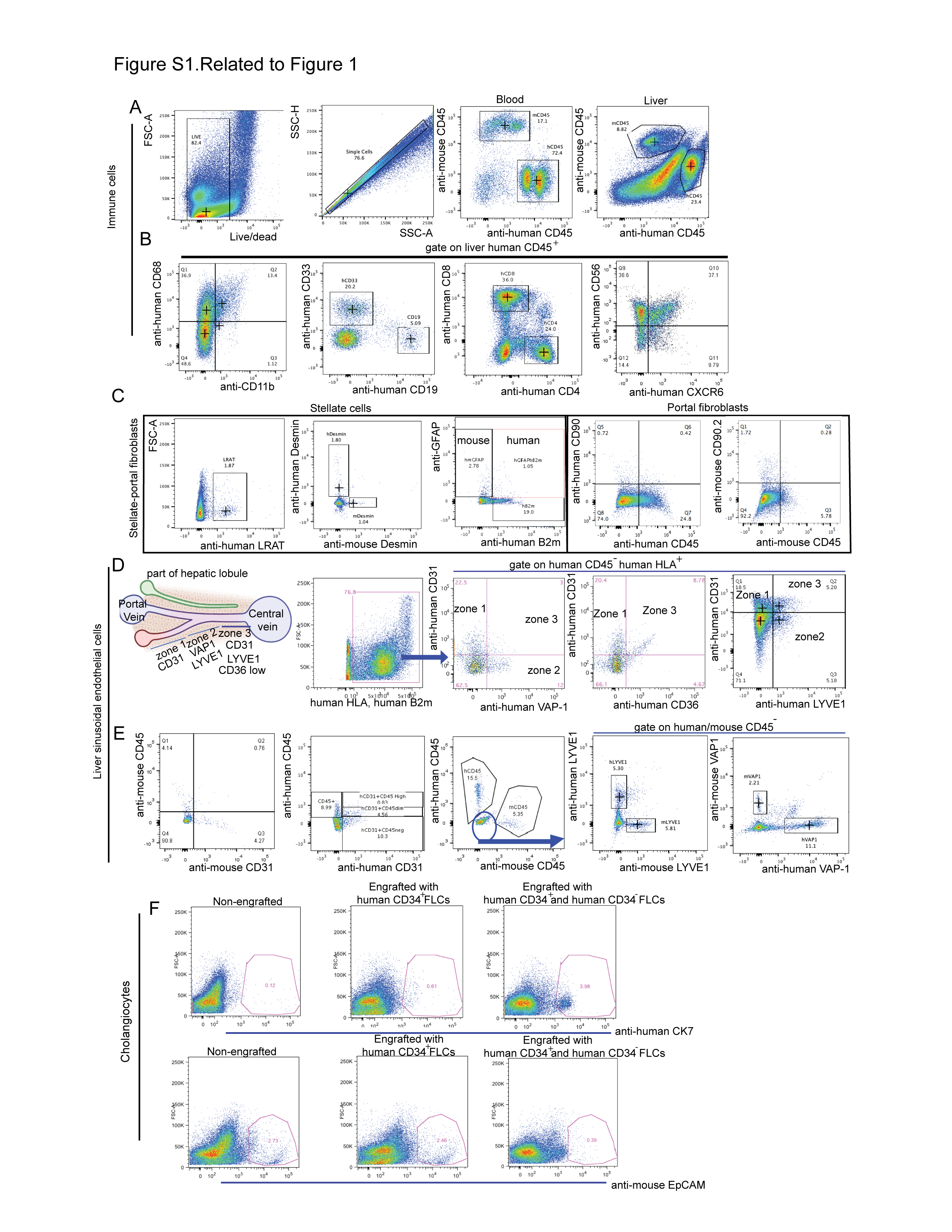

Supplement: 1 — Figure S1. Related to Figure 1: Representative flow cytometry plots used for quantification of human liver immune and non-immune cells. (A) Gating strategy for human and mouse immune cells of the blood and of the liver. (B) Human liver immune cell subtypes after gating to human CD45. (C) Human and mouse stellate cell and portal fibroblast-unique markers used for their quantification. (D) Subtypes of LSECs based on their location in the hepatic lobule (zone 1 cells are close to the portal vein, zone 2 cells are between the portal and the central vein and zone 3 cells are close to the central vein). The anti-human antibodies were tested for their human specificity with mouse controls and after gating on human HLA-A,B,C positive, human CD45 negative cells. (E) Gating strategy used to quantify human and mouse LSECs located in different zones. Since LSECs were shown to express CD4545, a common immune marker, we examined the expression of unique LSECs markers in the CD45 negative cells to avoid any contamination with immune cells. (F) Gating strategy used to quantify human (human CK7+) and mouse (mouse EpCAM+) cholangiocytes. The gates for human antibodies were put based on non-engrafted mice. [file NIHMS1922982-supplement-1.tif]

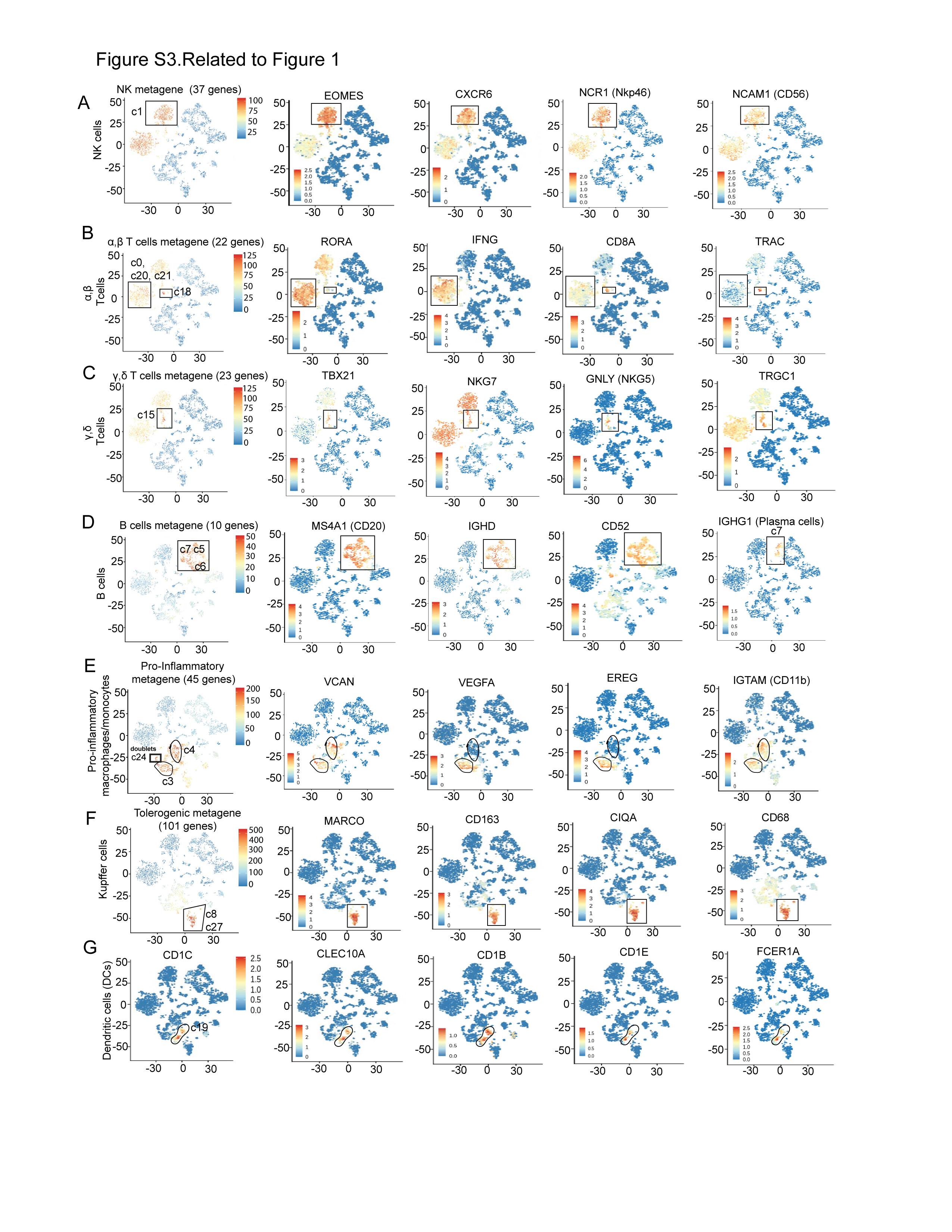

Supplement: 2 — Figure S2. Related to Figure 1: Human CD34+ FLCs support the same degree of NPC humanization in several models. (A) Liver humanization models employed, with or without liver damage. MISTRG6 mice were engrafted at a neonatal age with human CD34+ FLCs. These mice have mouse hepatocytes and human NPCs but no liver damage. Adult MISTRG-Fah−/− mice were treated with busulfan and on the next day were engrafted with human CD34+FLCs and mouse hepatocytes isolated from BALB/cJ donors or with human adult hepatocytes. NTBC was discontinued for 14 days to induce mouse hepatocyte death. Adult MISTRG6 mice were engrafted with human CD34+ FLCs and human adult hepatocytes after treatment with retrosine (two doses with a two-week interval), acetaminophen (APAP) and busulfan one day before human cell transplantation. One week after human hepatocyte engraftment, mice were treated with anti-mouse FAS weekly for 8 weeks to induce apoptosis in mouse hepatocytes only. These mice were named MISTRG6-RAF after the initials of the treatments used to help hepatocyte humanization (R: Retrosine, A: APAP, F: anti-FAS,). Cartoon was made using BioRender. (B-D) Percentage of humanization for B) liver immune cells, C) liver LSECs (VAP1+, CD31+, LYVE1+) and D) stellate cells (Desmin+ and GFAP+). (E) Percentage of human immune cells in the blood at different time points after human cell transplantation in MISTRG-Fah−/− mice. (F) Percentage of human immune cells in the blood. (G) Human plasma albumin measured with ELISA 12 weeks after human cell transplantation. (H) Immunofluorescence for human Hep Par1 in the liver of non-engrafted mice (mouse), in the healthy human liver, in MISTRG-Fah−/− and in MISTRG6-RAF mice engrafted with human hepatocytes and human CD34+ FLCs, 12 weeks after human cell transplantation. A whole lobe of the liver was scanned with a Keyence microscope. Mice were used at 12 weeks post-transplantation. Each dot in the graphs represents a biological replicate; n>4 biological replicat [file NIHMS1922982-supplement-2.tif]

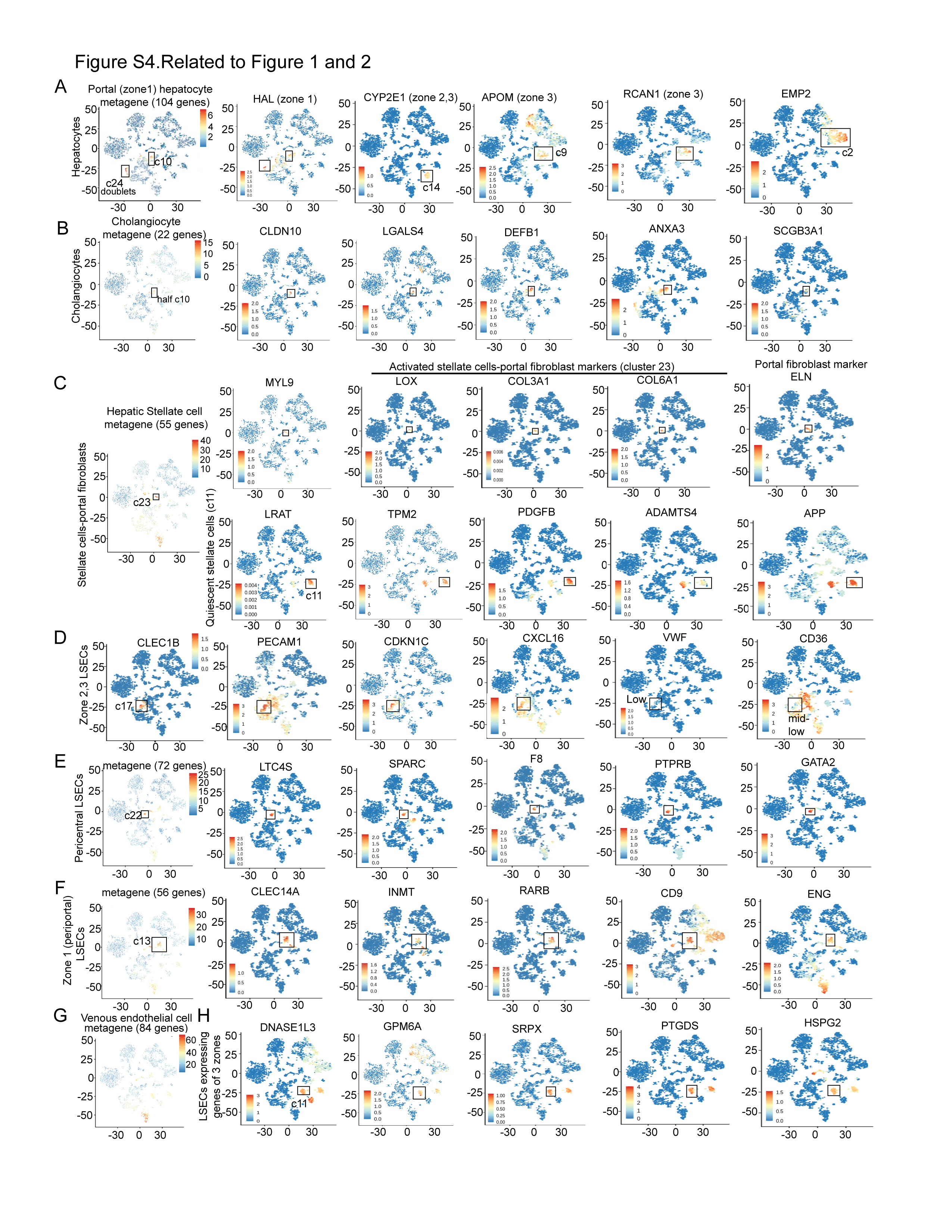

Supplement: 3 — Figure S3. Related to Figure 1: Human immune cell transcriptional signatures in the humanized liver.t-SNE plots displaying the expression of immune cell population metagenes and marker genes in the humanized liver single-cell dataset. Metagenes were calculated based upon public healthy human liver single-cell datasets24 and validated in other two datastes from human liver (human protein atlas24,46) for: (A) NK cells (c1). (B) αβ T-cells (c0, c20, c21, c18), c18 has several unique markers of MAIT cells. (C) Non-conventional γδ T-cells (c15). (D) B-cells (c5, c6), plasma cells (c7, c29). (E) Macrophages (c3) and monocytes (c4) with a signature called “pro-inflammatory” according to a public available healthy human liver single-cell dataset 24 c3 has enriched expression of “inflammatory” chemokine/cytokines and inflammasome components (CXCL2, CXCL8, CXCL9, CXCL10, CXCL11, CCL19, CCL20, IL1b, IL1a, NLRP3). The pro-inflammatory macrophage signature marks also a small cluster (c24) that express hepatocyte unique genes (Figure S4A). The latter population could potentially be explained by doublets consisting of macrophages and hepatocytes, or hepatocytes being phagocytosed. c4 is enriched on genes that are found in human liver monocytes in a recent publicly available single-nuclear/single-cell spatial dataset from non-perfused human livers 47. (F) Macrophages-Kupffer clusters (c8, c27) enriched in Kupffer cell genes according to 47 and mapped to the signature of the non-inflammatory/tolerogenic or immuno-modulatory liver macrophages (non-inflammatory macrophages according to24) (c8, c27). (G) Dendritic cells (DC) (c19). We didn’t have a signature from a healthy human liver to create a metagene but this cluster is enriched in conventional DC1 (cDC1) differentially expressed genes (DEGs) according to human liver single cell dataset 47 from patients with mixed pathologies (non-tumoural area of colorectal cancer liver metastasis, symptomatic cholecystolithiasis, obesity and Typ [file NIHMS1922982-supplement-3.tif]

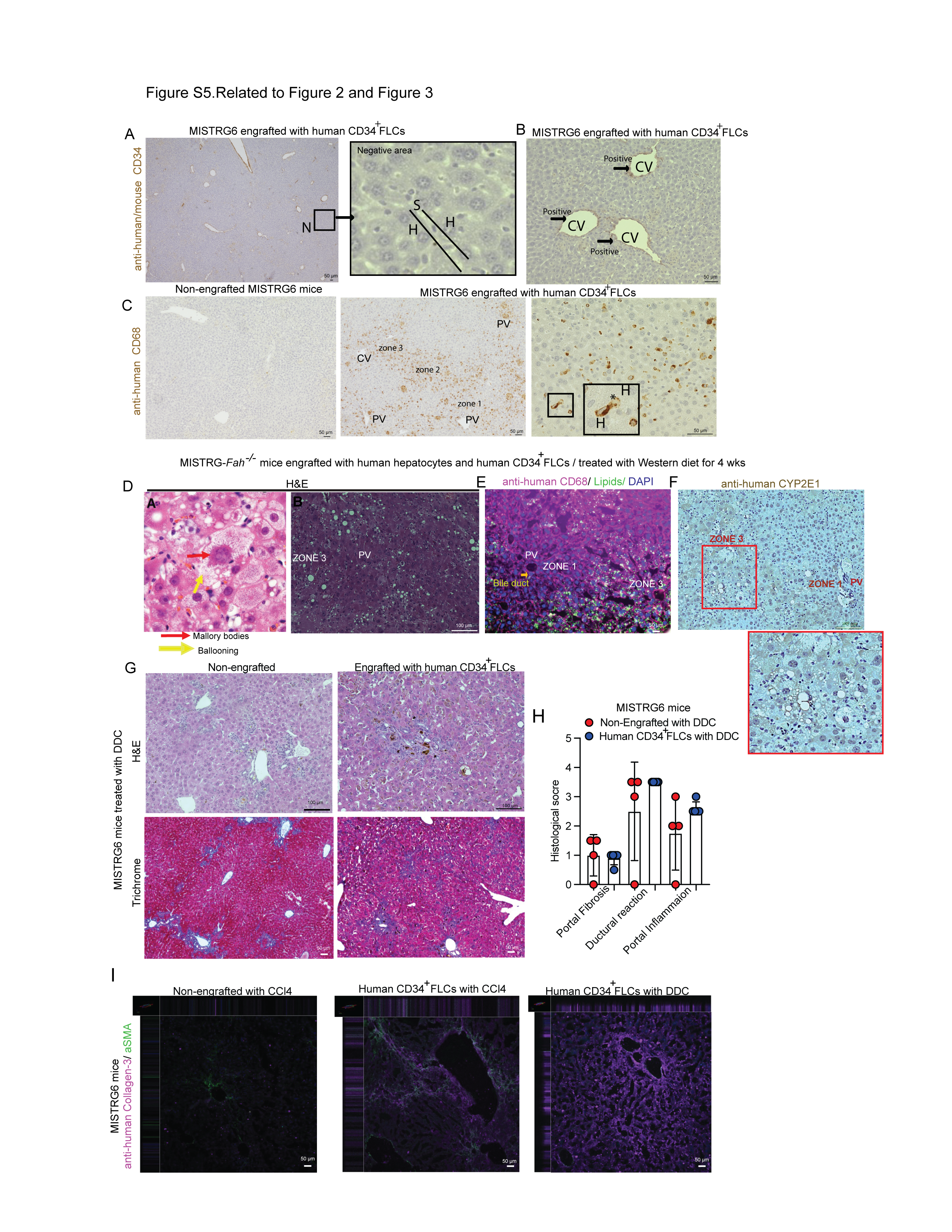

Supplement: 4 — Figure S4. Related to Figure 1 and 2: Human non-immune cell transcriptional signatures in the humanized liver. t-SNE plots displaying the expression of non-immune cell population metagenes and marker genes in the humanized liver single-cell dataset. Metagenes were calculated based upon 3 different public human liver single-cell datasets (human protein atlas24,46). (A) Human hepatocyte and zone-specific hepatocyte genes (zone 1 metagene and specific gene: c24 and half of c10, zone 2: c14, zone 3: c9). c24 is also expressing macrophage markers (Figure S3E) and possibly this cluster is doublets consisting of macrophages and hepatocytes, or hepatocytes being phagocytosed. c2 is expressing hepatocyte markers and markers of hepatocyte progenitors (CD24, SOX4). (B) Human cholangiocyte metagene and markers (half of c10). (C) Human hepatic stellate cell metagene and markers. Stellate cells are in two different clusters. One cluster expressing LRAT, which marks quiescent stellate cells48 (c11), and one cluster expressing COL3A1, LOX which marks activated stellate cells48 (c23). COL3A1, LOX, COL6A1 are also expressed by portal fibroblasts. ELN is expressed by portal fibroblasts and not by stellate cells in a normal liver only. Thus, c23 may include both activated stellate cells and portal fibroblasts. (D) Zone 2,3 LSECs (c17) are CLEC1B-high, VWF-low and co-express markers with macrophages (PECAM1, CXCL16, CD36). (E) Pericentral LSEC metagene and markers expressed by this zone (c22). (F) Zone 1 or periportal LSEC metagene and markers expressed by this zone (c13). (G) Venous endothelial cell metagene marks most LSEC clusters (c22, c12, c13) as in human liver LSECs (human protein atlas24,46) but is not enriched in any cluster indicating possibly the absence of human venous endothelial cells in our dataset. (H) Markers of LSECs not specific for a zone. Predominant venous endothelial gene (PTGDS) and LSECs genes are expressed mainly on c12. c11 (stellate cell cluster) has several [file NIHMS1922982-supplement-4.tif]

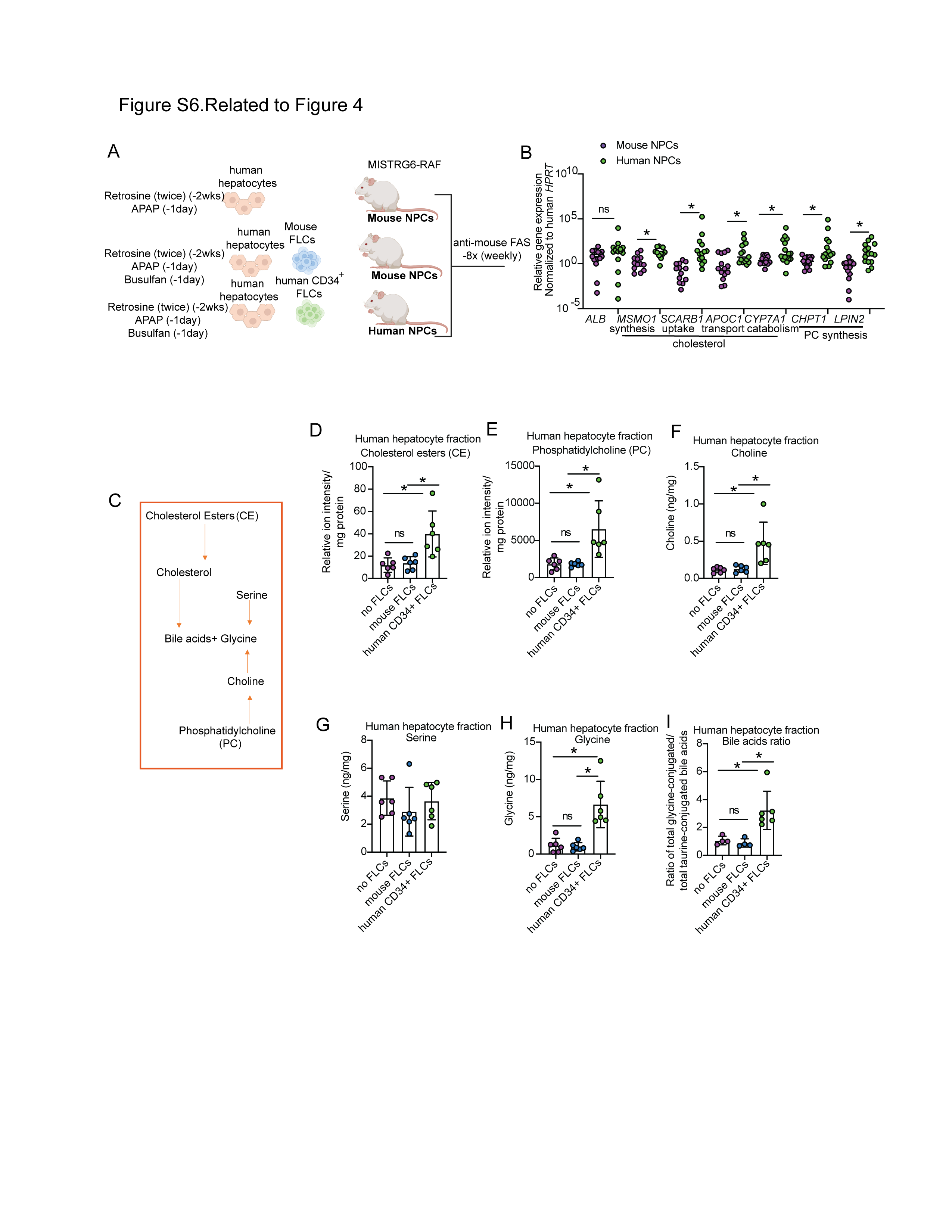

Supplement: 5 — Figure S5. Related to Figure 2 and Figure 3: Immunohistochemical analysis. (A) Immunohistochemistry for CD34 (human and mouse, dual specificity), a marker of fetal/immature LSECs and venous endothelial cells. A negative area (N), including sinusoids (S) and the surrounding hepatocytes (H) is shown at a higher magnification. The lack of CD34 in sinusoidal endothelial cells located between hepatocytes indicates their maturity. (B) Venous endothelial cells (black arrows) are positive for CD34, as expected in the adult liver. (C) Immunohistochemistry for human CD68, a macrophage marker. Human macrophages are detected in all three zones of the liver (zone 1, zone 2, zone 3). The lack of human CD68 staining in the liver of mice non-engrafted with human cells confirms the human specificity of the antibody. Human CD68+ cells are located between hepatocytes (cells with big nuclei, indicated with H) in the sinusoids (indicated with S). All livers were collected from MISTRG6-RAF mice 12 weeks after human CD34+ FLC and human hepatocyte transplantation. (D) H&E showing A) Mallory bodies (red arrow) ballooning (yellow arrow) and B) steatotic hepatocytes enriched in zone 3 but not in zone 1 that is the area close to porta vein (PV). (E) Immunofluorescence for human CD68 (in red) and staining with BODIPY for lipids (in green) and counterstaining with DAPI in blue. (F) IHC for human Cyp2E1(zone 2–3 hepatocyte marker) shows human steatotic hepatocytes in the zone 3 area. (G) H&E and trichrome staining in engrafted and non-engrafted MISTRG6 mice treated with DDC. (H) Histological score for ductular reaction, portal inflammation and portal fibrosis in engrafted and non-engrafted MISTRG6 mice treated with DDC. (I) IHC for human collagen-3 (in purple) and aSMA (in green) in mice treated with CCl4 or DDC. The lack of human collagen-3 staining in the liver of mice that were not engrafted with human cells confirms the human specificity of the antibody. [file NIHMS1922982-supplement-5.tif]

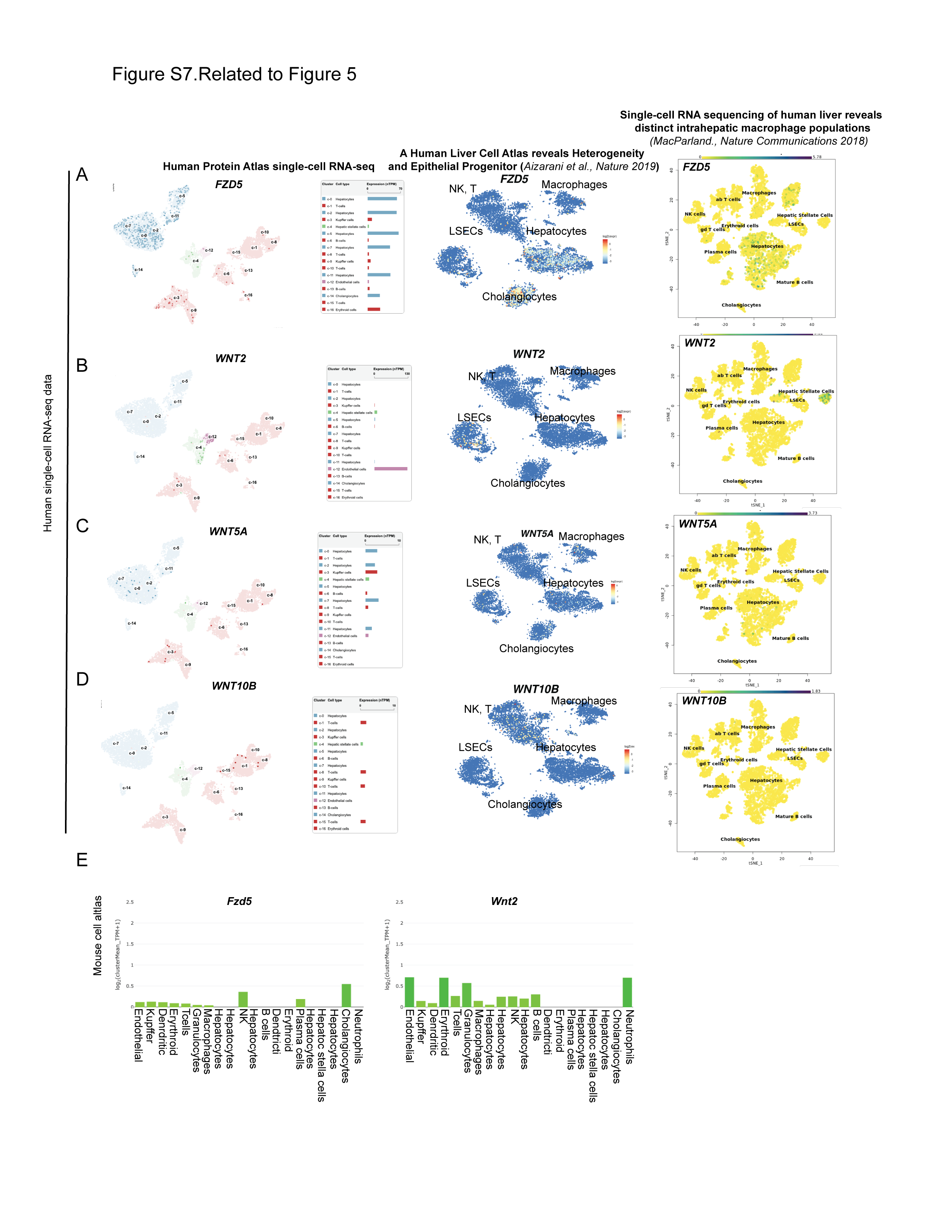

Supplement: 6 — Figure S6. Related to Figure 4: Human NPCs shape hepatocyte metabolism in MISTRG6-RAF mice. (A) Adult MISTRG6-RAF mice were engrafted with human hepatocytes only or with human hepatocytes with mouse FLCs (gestational age 15 days) or with human CD34+ FLCs after treatment with retrosine and APAP. The mouse or human FLC group also received busulfan 1 day before transplantation. Following anti-mouse FAS antibody treatment weekly for 8 weeks, the mice were euthanized 4 weeks after the last anti-FAS treatment. Cartoon was made using BioRender. (B) RT-qPCR in the hepatocyte fraction of MISTRG-RAF mice receiving human hepatocytes alone (mouse NPC group) or together with human CD34+ FLCs (human NPC group). We examined genes involved in cholesterol synthesis (MSMO1), uptake (SCARB1), transport (APOC1), catabolism to bile acids (CYP7A1) or PC synthesis (CHPT1 and LPIN2). (C-I) Metabolites measured by HPLC-MS/MS in all groups in the human hepatocyte fraction include: D) Total cholesterol esters (CE), E) Total phosphatidylcholine (PC), F) Choline, G) Serine, H) Glycine, I) ratio of total glycine to total taurine-conjugated bile acids. Each dot in the graphs represents a biological replicate; n>3 biological replicates from 2 independent experiments. Data represent mean ± SEM. ns: non-significant, *p < 0.05. [file NIHMS1922982-supplement-6.tif]

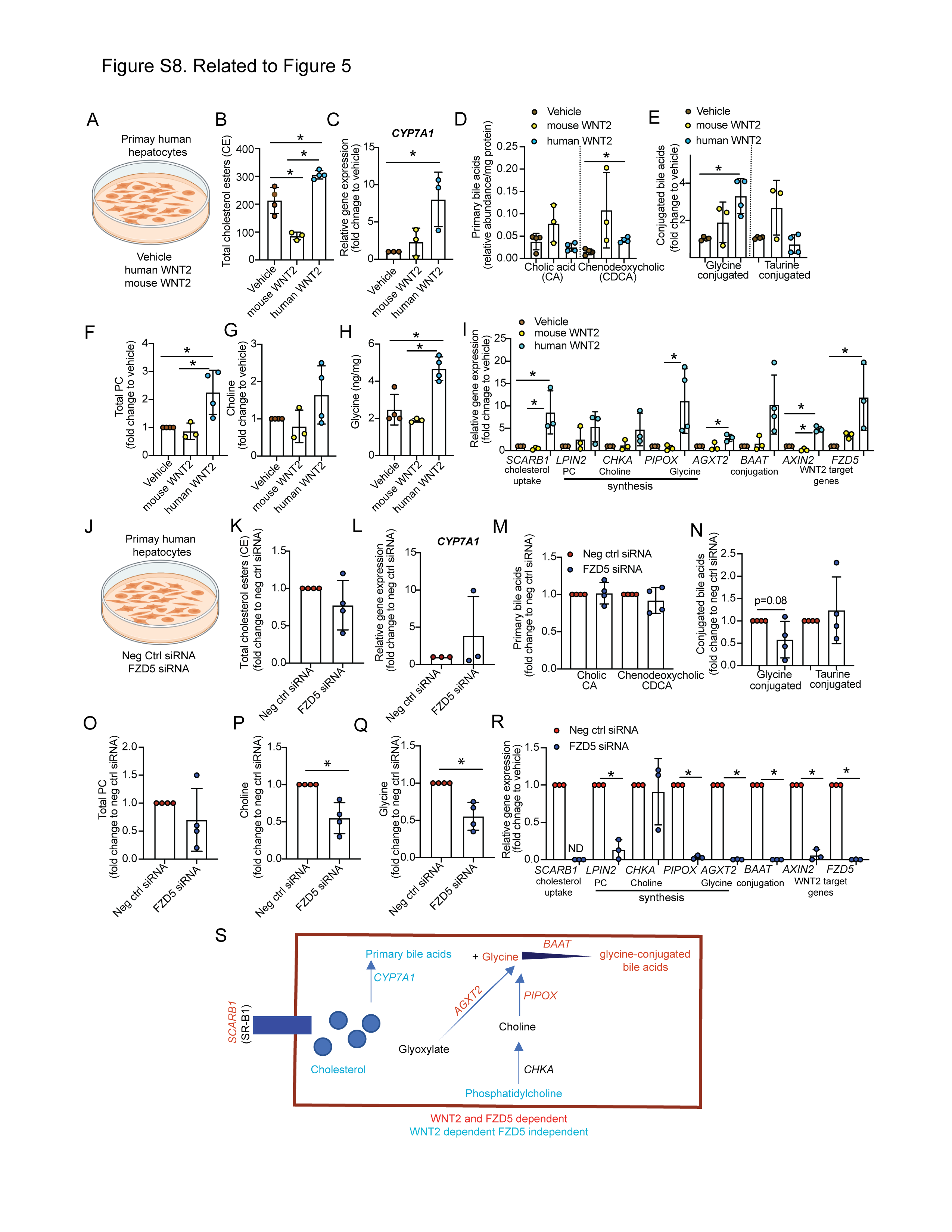

Supplement: 7 — Figure S7. Related to Figure 5: Species differences in the gene expression of FZD5 and its ligands between human and mouse liver. (A) FZD5, (B) WNT2, (C) WNT5A, (D) WNT10B gene expression in the human liver by single-cell RNA-seq from three different datasets (human protein atlas24,46). (E) Fzd5 and Wnt2 gene expression in the mouse liver by single-cell RNA-seq. Data retrieved from the mouse cell atlas. [file NIHMS1922982-supplement-7.tif]

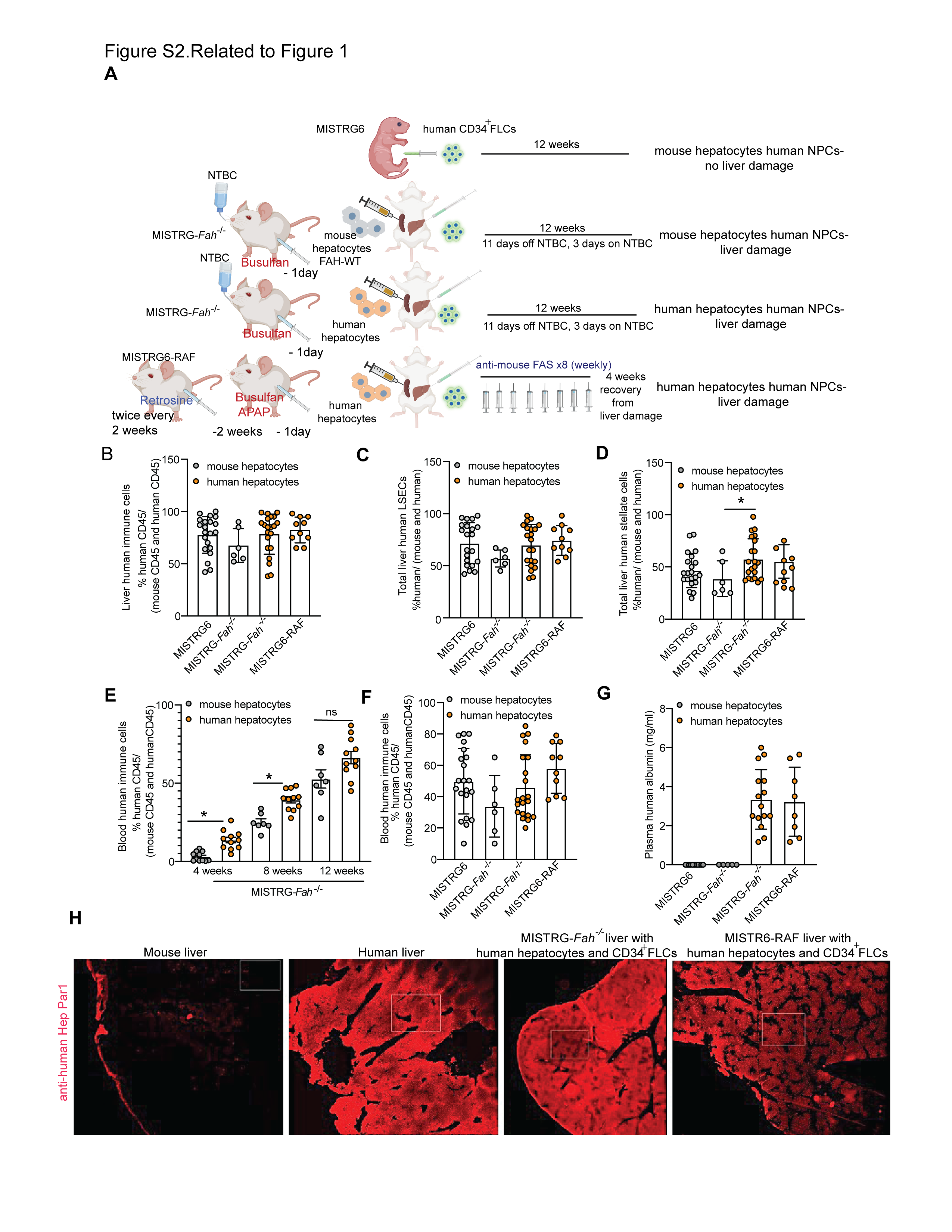

Supplement: 8 — Figure S8. Related to Figure 5: FZD5-dependent and FZD5-independent WNT2-mediated effects on human hepatocytes. (A) Cultured primary human hepatocytes were treated with human or mouse WNT2 or its vehicle (DMSO) for 24 hours. (B) Total cholesterol esters (CE) measured by HPLC-MS/MS. (C) CYP7A1 relative gene expression. (D-H) HPLC-MS/MS analysis for D) primary non-conjugated bile acids (CA and CDCA), E) primary total glycine or taurine-conjugated bile acids, F) total phosphatidylcholine (PC), G) choline and H) glycine. (I) Relative expression of genes involved in cholesterol uptake (SCARB1), PC synthesis (LPIN2), choline synthesis (CHKA), glycine synthesis (AGXT2, PIPOX), bile acid conjugation (BAAT) and of WNT2 target genes (AXIN2, FZD5) by RT-qPCR, displayed as fold-change to vehicle. (J-R) Cultured primary human hepatocytes were transfected with FZD5 siRNA or negative control (Neg ctrl) for 24 hours. HPLC-MS/MS and RT-qPCR analyses were performed for: K) total cholesterol esters (CE), L) Cyp7A1 relative gene expression, M) primary non-conjugated bile acids (CA and CDCA), N) primary total glycine or taurine-conjugated bile acids, O) total phosphatidylcholine (PC), P) choline, Q) glycine, R) genes involved in cholesterol uptake (SCARB1), PC synthesis (LPIN2), choline synthesis (CHKA), glycine synthesis (AGXT2, PIPOX), bile acid conjugation (BAAT) or WNT2 target genes, (AXIN2, FZD5). In K-R data are shown as fold change to vehicle. (S) Schematic summary of FZD5-dependent and FZD5-independent effects of WNT2 on primary human hepatocytes. Each dot in the graphs represents a biological replicate; Data represent mean ± SEM. *p < 0.05. [file NIHMS1922982-supplement-8.tif]
